# Effects of daily mean temperature and other meteorological variables on bacillary dysentery in Beijing-Tianjin-Hebei region, China

**DOI:** 10.1265/ehpm.21-00005

**Published:** 2022-03-19

**Authors:** Qinxue Chang, Keyun Wang, Honglu Zhang, Changping Li, Yong Wang, Huaiqi Jing, Shanshan Li, Yuming Guo, Zhuang Cui, Wenyi Zhang

**Affiliations:** 1 Department of Epidemiology and Biostatistics, School of Public Health, Tianjin Medical University, Heping District, Tianjin, 300070, P.R. China; 2 Chinese PLA Center for Disease Control and Prevention, Beijing, 100071, P.R. China; 3 State Key Laboratory of Infectious Disease Prevention and Control, National Institute for Communicable Disease Control and Prevention, Chinese Center for Disease Control and Prevention, Beijing, 102206, P.R. China; 4 Department of Epidemiology and Preventive Medicine, School of Public Health and Preventive Medicine, Monash University, Melbourne, Australia

**Keywords:** Distributed lag non-linear model, Generalized additive model, Nonlinear and interaction effects, Bacillary dysentery

## Abstract

**Background:**

Although previous studies have shown that meteorological factors such as temperature are related to the incidence of bacillary dysentery (BD), researches about the non-linear and interaction effect among meteorological variables remain limited. The objective of this study was to analyze the effects of temperature and other meteorological variables on BD in Beijing-Tianjin-Hebei region, which is a high-risk area for BD distribution.

**Methods:**

Our study was based on the daily-scale data of BD cases and meteorological variables from 2014 to 2019, using generalized additive model (GAM) to explore the relationship between meteorological variables and BD cases and distributed lag non-linear model (DLNM) to analyze the lag and cumulative effects. The interaction effects and stratified analysis were developed by the GAM.

**Results:**

A total of 147,001 cases were reported from 2014 to 2019. The relationship between temperature and BD was approximately liner above 0 °C, but the turning point of total temperature effect was 10 °C. Results of DLNM indicated that the effect of high temperature was significant on lag 5d and lag 6d, and the lag effect showed that each 5 °C rise caused a 3% [Relative risk (RR) = 1.03, 95% Confidence interval (CI): 1.02–1.05] increase in BD cases. The cumulative BD cases delayed by 7 days increased by 31% for each 5 °C rise in temperature above 10 °C (RR = 1.31, 95% CI: 1.30–1.33). The interaction effects and stratified analysis manifested that the incidence of BD was highest in hot and humid climates.

**Conclusions:**

This study suggests that temperature can significantly affect the incidence of BD, and its effect can be enhanced by humidity and precipitation, which means that the hot and humid environment positively increases the incidence of BD.

**Supplementary information:**

The online version contains supplementary material available at https://doi.org/10.1265/ehpm.21-00005.

## Graphical Abstract

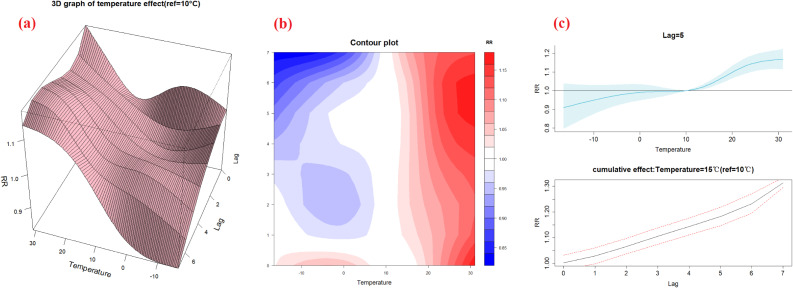

## 1 Introduction

Bacillary dysentery (BD), also known as shigellosis, is an intestinal infectious disease caused by *Shigella*, which is transmitted mainly through fecal-oral contact, the consumption of contaminated food or water, or contact with an infected person [1–4]. Whether in developed or developing countries, BD is a major public health threat. It is estimated that the annual incidence of BD worldwide is approximately 165 million, with more than 1 million deaths each year [5]. In China, although the morbidity of BD has declined in the past few decades because of rapid improvements in water supply and sanitation [6], BD remains the third most notifiable infectious disease, especially in remote and underdeveloped regions [7].

The incidence of BD is determined by a combination of factors, including dietary lifestyle, climate change, sociocultural and economic development and geographic condition. Of these, climatic variability, which affects the incubation period of *Shigella*, is believed to be a major factor affecting transmission [8]. Recently, the role of meteorological factors on the incidence of BD has received much attention. Although the specific mechanism is unclear, a number of studies have shown that meteorological factors play an important role in the spread of BD [9–11]. For example, a study in Chongqing used a support vector regression model to show that temperature, air pressure, precipitation and sunshine could affect the growth, survival and transmission of enteric bacteria in the environment [10].

Other studies, both within and outside China, have usually used weekly or monthly incidence and meteorological data to evaluate BD transmission [12–16]. However, as the time scale was wide and the time span was short, these studies could not accurately model the impact of meteorological factors on BD. In fact, the effect of meteorological factors on human health is non-linear (but rather either J, V or U-shaped), and there exist lag effects and the interaction between meteorological factors [17]. Unfortunately, there is currently little quantitative data on the effect of meteorological factors on the incidence of BD, especially on the lag effect on BD and the interaction between meteorological factors.

The Beijing-Tianjin-Hebei region is the biggest urban agglomeration in northern China and is one of the country’s BD hot spots [18, 19]. So it is a high-risk area for BD and has a high BD disease burden [20]. According to the China National Disease Surveillance System, the highest annual mean BD incidence rates are usually observed in Beijing and Tianjin [15, 18, 21]. Nevertheless, in this region, few studies have been conducted to explore the effect of daily meteorological variables on the incidence of BD. Our study, therefore, explored the effects of temperature and other meteorological variables on BD. We used daily-scale BD incidence data of the Beijing-Tianjin-Hebei region and distributed lag non-linear model (DLNM) [22] to evaluate the effects of environmental temperature hysteresis and the interaction with other meteorological variables on BD. Our results will provide insight into the factors that influence the incidence of BD and may lead to timely predictions of BD outbreaks in the Beijing-Tianjin-Hebei region.

## 2 Material and methods

### 2.1 Data collection

Daily reports of BD cases between January 2014 and December 2019 in the Beijing-Tianjin-Hebei region were obtained from Chinese Center for Disease Control and Prevention, and the meteorological data over the same period were obtained from China Meteorological Science Data Sharing Service System, which included mean temperature, relative humidity, air pressure, sunshine duration, wind speed and precipitation, etc. For municipality with two or more meteorological stations, we used the average value of multiple stations to represent the weather conditions of the municipality.

### 2.2 Statistical analysis

Firstly, the reported data on BD cases and meteorological data were cleaned and sorted into daily-scale time series data, which were descriptively analyzed to evaluate their characteristics and distribution.

Secondly, as the incidence of BD was considered to be a small probability event compared with the population of the study area, it was approximated to a Poisson distribution. Therefore, a generalized additive model (GAM) with quasi-Poisson regression, to account for over-dispersion, was applied to explore the relationship between the meteorological variables and the number of BD cases. To avoid multicollinearity, Spearman’s correlation coefficients were initially used to explore the interrelationships between the meteorological variables [23]. Based on the results, daily mean temperature, relative humidity, sunshine duration, precipitation and daily mean wind speed were then incorporated into the final model.

A distributed lag non-linear model (DLNM) combined with GAM was developed to analyze the non-linear relationship, the lag effect and the cumulative effect of the mean temperature on BD, and to obtain estimates of the temperature-BD associations. We established a cross-basis matrix for temperature, with daily incidence as the dependent variable and natural cubic spline as the basis function in the exposure dimension, with nodes set at the 25th, 50th and 75th percentiles of the mean temperature. A polynomial function was used in the lag dimension, with the lagging degree of freedom set at 3 according to the Quasi Akaike Information Criterion. The incubation period of BD is generally 2–3 days, but can be up to 7 days [24], and the impact of meteorological variables on pathogens is mostly short-term; therefore, this study set the maximum lagging period to 7 days. The model can be specified as:

$$\text{log}[E(Y_{t})]=\beta +\sum_{k=0}^{7}\alpha_{k}{\text{temp}}_{t-k}+s(\text{weather})+s(\text{time})$$

Where *E*(*Y_t_*) refers to the expected daily BD cases on day t, *β* is the intercept; *α_k_* is the effect estimate of the temperature *k* days before the onset day of illness. *s*( ), the thin plate spline function, are applied to adjust for other meteorological variables, long-term and seasonal trends, and its degree of freedom is automatically selected based on the integral generalized cross validation method.

We expressed the relative risk (RR) of BD infection with a 95% confidence interval (CI) of exposure-response at different temperatures, using the turning point of the temperature effect as reference [25]. All models were fitted in R (version 4.0.3) with “dlnm” and “mgcv” packages, and a two-sided test was adopted with the test level α = 0.05.

## 3 Results

### 3.1 Descriptive analysis

**Fig. 1:**
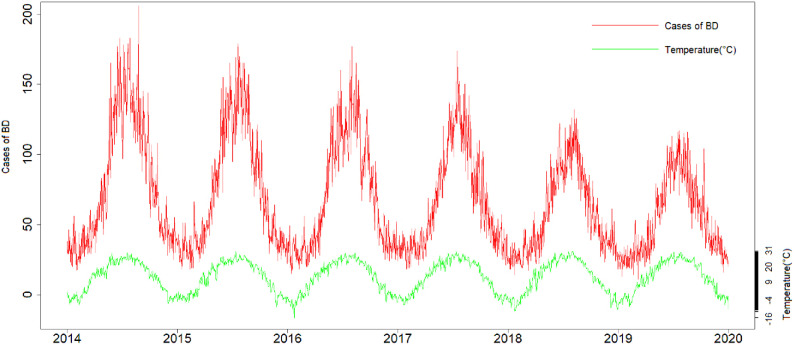
The time-series distributions of BD and mean temperature in Beijing-Tianjin-Hebei region. From the peak of the cases of BD and the mean temperature, there is a certain lag effect between them.

A total of 147,001 cases of BD were reported in the Beijing-Tianjin-Hebei region from 2014 to 2019, with an average daily case of 67.09 ± 38.28. The average values for daily mean temperature, relative humidity, sunshine duration, precipitation and mean wind speed were 12.32 °C, 56.15%, 6.82 h, 4.37 mm and 2.11 m/s, respectively (Table 1). Figure 1 shows the time-series distributions of BD cases and mean temperature, clearly demonstrating both the periodicity and the seasonality of BD cases. While the incidence of BD in the Beijing-Tianjin-Hebei region has declined over the past two years, most cases were occurred in the summer and fall (June–September), with peaks in July and August. Moreover, as shown in the figure, the time trend of daily BD cases was positively associated with daily mean temperature.

**Table 1:** Summary statistic for daily meteorological variables and the number of BD cases in Beijing-Tianjin-Hebei region.

	**Mean ± SD**	**Minimum**	***P*_25_**	**Median**	***P*_75_**	**Maximum**
Cases of BD	67.09 ± 38.28	11	35	55	95	206
Mean temperature (°C)	12.32 ± 11.35	−16.5	1.0	13.7	22.9	31.4
Relative humidity (%)	56.15 ± 17.23	13	42	56	70	95
Sunshine duration (h)	6.82 ± 3.45	0	4.27	7.57	9.44	13.23
Precipitation (mm)	1.42 ± 4.51	0	0	0	0.7	115.2
Wind speed (m/s)	2.11 ± 0.70	0.87	1.60	1.97	2.47	5.80

Abbreviations: SD, standard deviation; *P_x_*, xth percentile.

### 3.2 Preliminary relationship between meteorological variables and BD

**Fig. 2:**
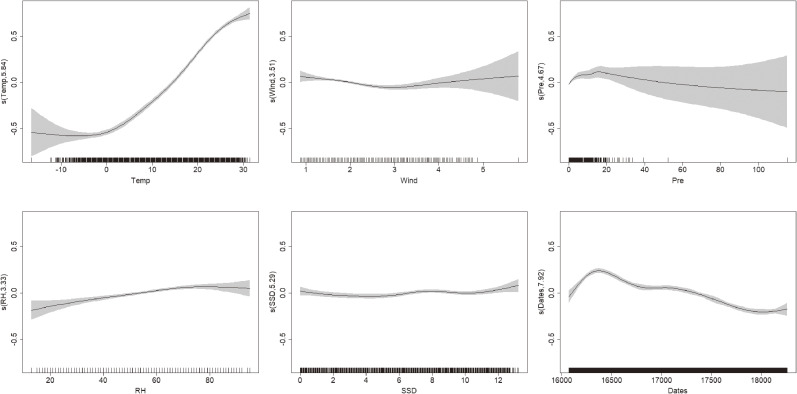
The relationship curves between meteorological variables and BD. The daily mean temperature had a nonlinear effect on BD and showed an approximately linear trend above 0 °C. Abbreviations: Temp, daily mean temperature; Wind, daily mean wind speed; Pre, precipitation; RH, relative humidity; SSD, sunshine duration.

A generalized additive model was established to fit the association between the meteorological variables and the number of BD cases. The daily mean temperature had a nonlinear effect on BD, but showed an approximately linear trend above 0 °C (Fig. 2), which indicated that the single threshold of the influence of temperature on BD was 0 °C. In addition, the weak influence of daily mean wind speed and relative humidity was also nonlinear. There was a weak inverse relationship between precipitation and BD, but the effect of sunshine duration was negligible. Exploratory analyses of the three areas individually (Beijing, Tianjin and Hebei) obtained similar results (see Fig. S1–S3). Based on these results, a distribution lag non-linear model was, therefore, established.

### 3.3 Effect of daily mean temperature on BD incidence

**Fig. 3:**
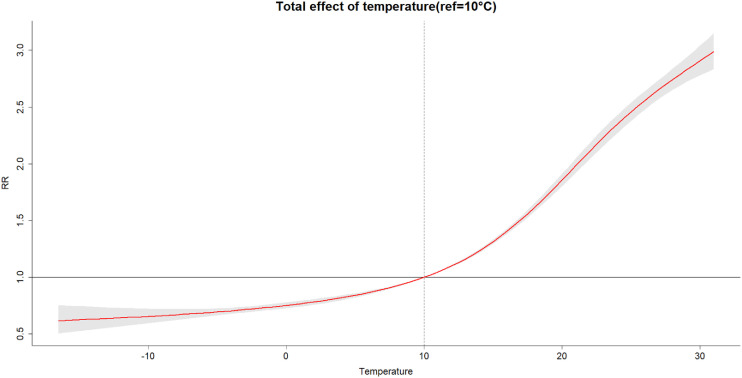
The total effect of daily mean temperature. The risk of BD had a hardly increase when the temperature was lower than 10 °C, while it caused a substantial increase when higher than 10 °C.

**Fig. 4:**
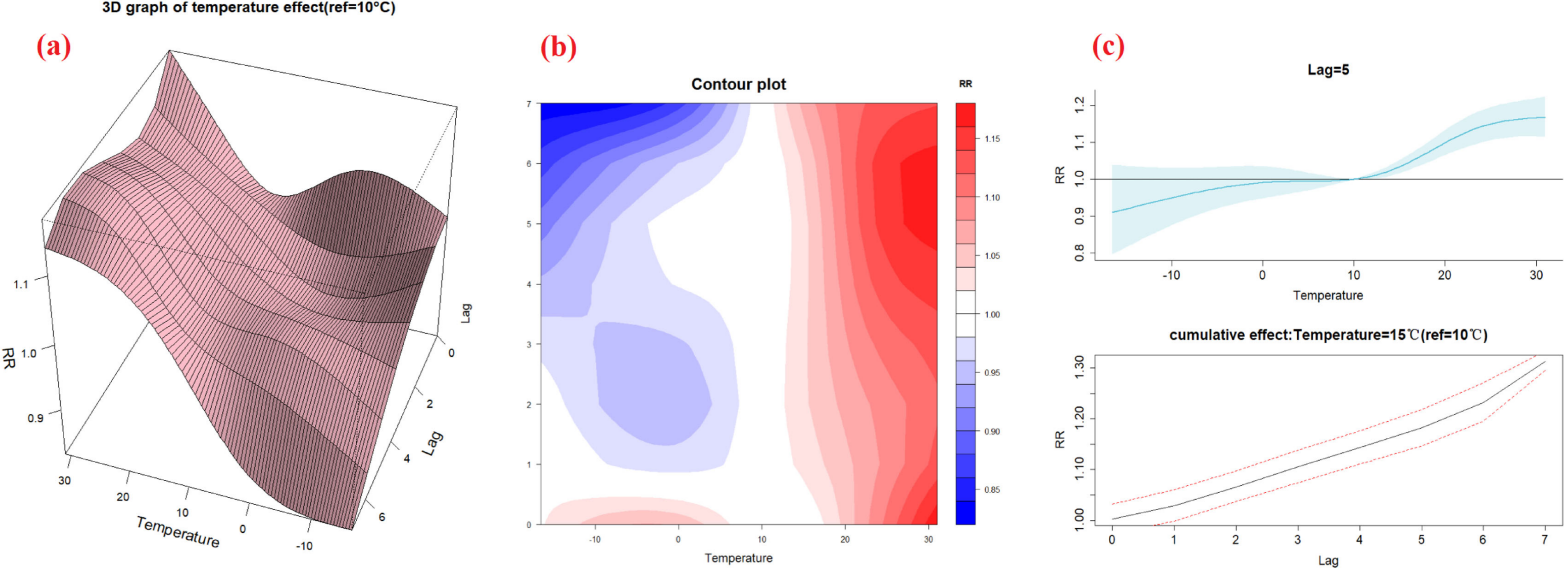
The visualization of the lag effect. The 3D graph (a) and contour plot (b) of lag effect show that the lag effect is strongest on the 5th and 6th days. The concrete lag effect on lag 5d and cumulative effect in 15 °C (c) indicate that BD cases on lag 5d increased by 3% and the cumulative BD cases delayed by 7 days increased by 31% for each 5 °C rise.

Figure 3 shows the overall effect of daily mean temperature on BD incidence. When the daily mean temperature was below 10 °C, there was little increase in the relative risk of BD infection, while when it was higher than 10 °C, there was a significant increase in the risk of disease. Therefore, in the Beijing-Tianjin-Hebei region, 10 °C was the turning point of total temperature effect, which was the same for each of the three regions when assessed separately (see Fig. S4–S6). The lag effect, based on a reference temperature of 10 °C, confirmed that on lag day 5 (lag 5d) and lag 6d, the higher the temperature, the greater the relative risk of BD infection (Fig. 4). Using P_2.5_ and P_97.5_ as the reference values for the cold and heat effects showed that, when the daily mean temperature was 28 °C, the number of BD cases on lag 5d increased by 16% (RR = 1.16, 95% CI: 1.12–1.21) (Table 2). Results of lag effect showed that each 5 °C rise in temperature caused a 3% (RR = 1.03, 95% CI: 1.02–1.05) increase in the number of cases of BD on lag 5d. Analysis of the cumulative effect showed that the cumulative number of BD cases delayed by 7 days increased by 31% for each 5 °C rise in temperature above 10 °C (RR = 1.31, 95% CI: 1.30–1.33) (Fig. 4).

**Table 2:** The relative risk of lag effect of different temperatures and lag days on the onset of BD.

	***P_2.5_*: −7 °C**	***P_25_*: 1 °C**	***P_50_*: 14 °C**	***P_75_*: 23 °C**	***P_97.5_*: 28 °C**
Lag0	1.057 (0.952–1.174)	1.051 (0.984–1.123)	0.998 (0.975–1.023)	1.083^*^ (1.014–1.156)	1.142^*^ (1.059–1.23)
Lag1	0.976 (0.93–1.023)	0.969^*^ (0.94–0.998)	1.021^*^ (1.01–1.032)	1.073^*^ (1.044–1.103)	1.106^*^ (1.073–1.14)
Lag2	0.951 (0.89–1.015)	0.947^*^ (0.91–0.987)	1.029^*^ (1.015–1.044)	1.08^*^ (1.04–1.122)	1.103^*^ (1.057–1.152)
Lag3	0.956 (0.909–1.005)	0.96^*^ (0.93–0.99)	1.029^*^ (1.018–1.04)	1.098^*^ (1.067–1.13)	1.12^*^ (1.084–1.157)
Lag4	0.968 (0.921–1.018)	0.983 (0.952–1.014)	1.025^*^ (1.014–1.036)	1.118^*^ (1.088–1.15)	1.144^*^ (1.109–1.18)
Lag5	0.966 (0.904–1.031)	0.992 (0.953–1.033)	1.024^*^ (1.009–1.039)	1.135^*^ (1.093–1.178)	1.164^*^ **(1.116–1.212)**
Lag6	0.926^*^ (0.883–0.972)	0.964^*^ (0.936–0.994)	1.032^*^ (1.021–1.043)	1.138^*^ (1.107–1.171)	1.164^*^ **(1.128–1.202)**
Lag7	0.835^*^ (0.751–0.927)	0.88^*^ (0.824–0.941)	1.055^*^ (1.03–1.08)	1.123^*^ (1.053–1.196)	1.135^*^ (1.058–1.218)

^*^P < 0.05. The lag effect was based on a reference temperature of 10 °C. The bold text means that the risk of BD with an increase of 16.4% is highest on lag 5d and lag 6d when the daily mean temperature is 28 °C.

### 3.4 Interaction effects and stratified analysis

**Fig. 5:**
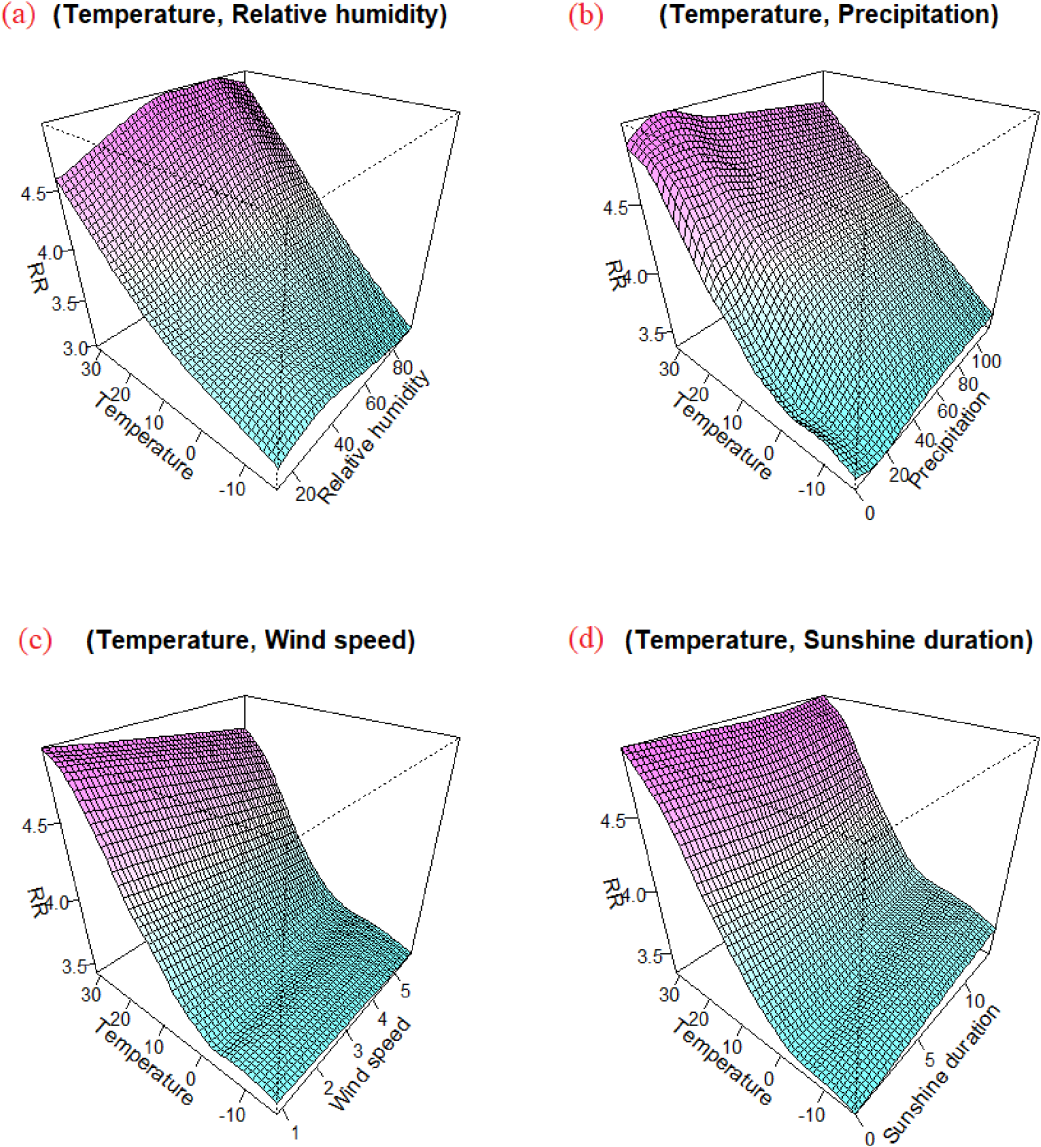
The modification and stratified analysis by other meteorological variables on the effect of mean temperature. The modification effect of relative humidity on temperature is positive (a), and that of wind speed is reverse (c). There is a positive relationship between the incidence of BD and precipitation during 0∼20 mm and reverse relationship with precipitation exceeding 20 mm (b). In our research, the precipitation in the Beijing-Tianjin-Hebei region was defined as the total precipitation of these three places, and its distribution was significantly left skewed, concentrate in 0∼20 mm. In the total of 2191 days from 2014 to 2019, the number of days with precipitation exceeding 20 mm was 138 days, accounting for only 6%. Thus, the number of BD cases with precipitation exceeding 20 mm was not representative, and to a certain extent could not represent the incidence of BD in heavy precipitation. However, the risk of BD with precipitation is still higher than that without precipitation.

**Fig. 6:**
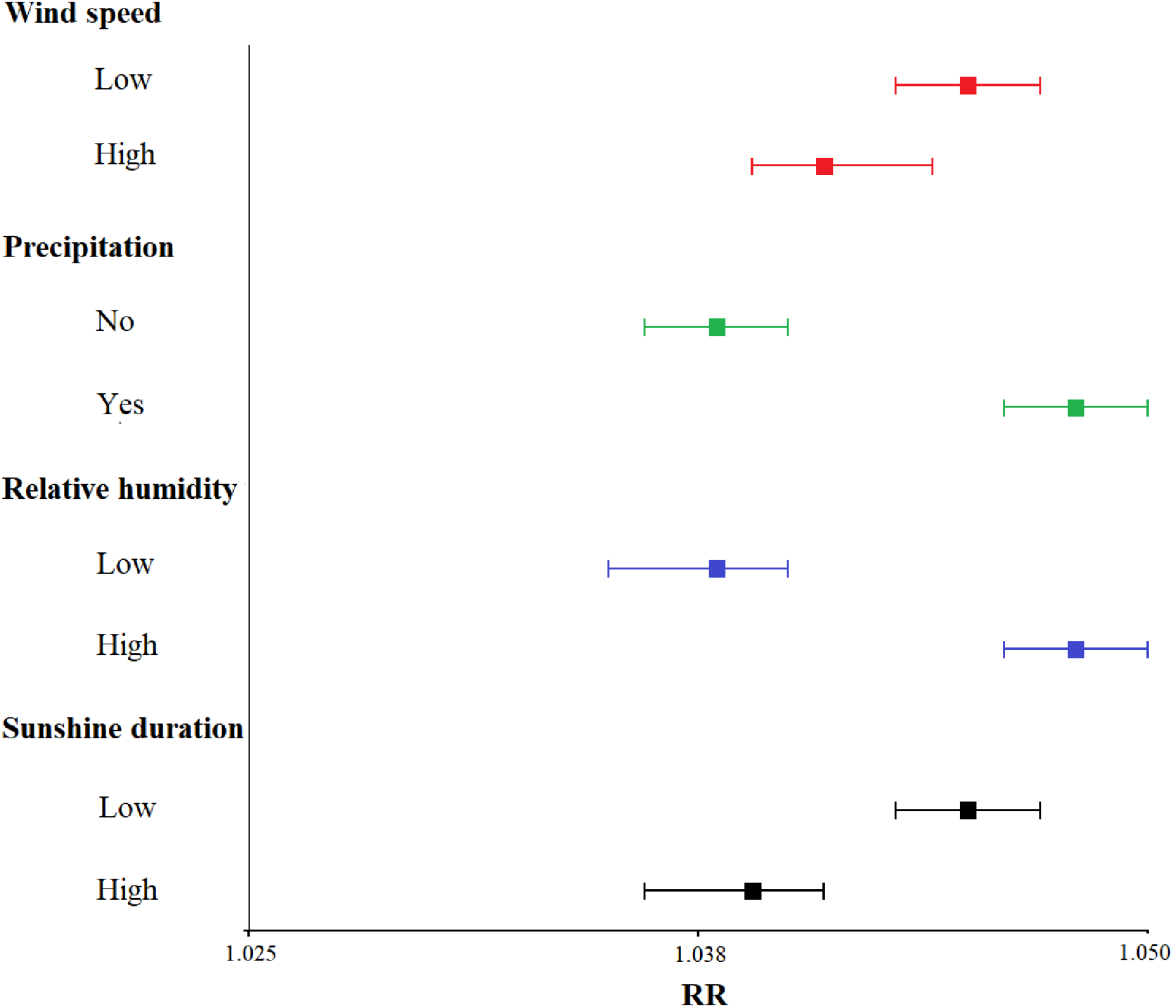
Relative risk (RR) stratified by wind speed, precipitation, relative humidity and sunshine duration. Squares represent the RR and error bars indicate the 95% CI.

Figure 5 illustrates the modification by other meteorological variables on the effect of temperature on BD. The modification effect suggested the relative risk of BD infection was greater in a climate with higher temperature and higher humidity or a condition of high temperatures with a little precipitation or low wind speed. Figure 6 shows the effect of daily mean temperature on the relative risk of BD infection stratified by other meteorological variables. The stratified analyses showed that high humidity and rainy weather enhanced the effect of temperature on BD, while the function of wind speed and sunshine duration was opposite.

### 3.5 The lag effect in three separate regions

**Fig. 7:**
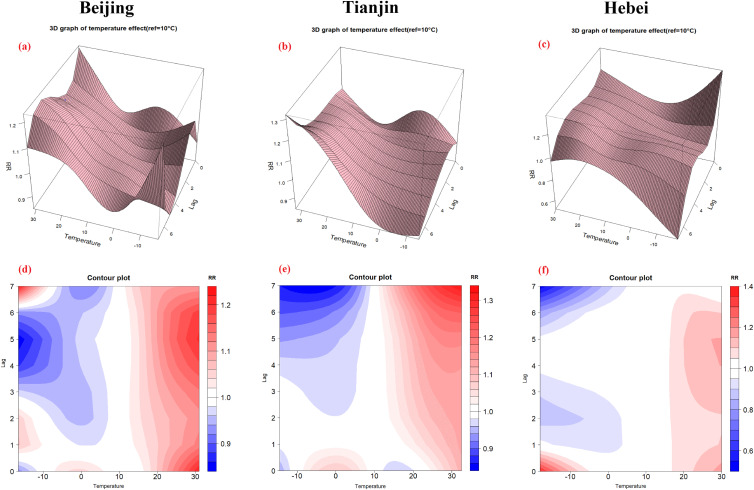
The 3D graph (a∼c), contour plot (d∼f) of lag effect in Beijing, Tianjin and Hebei, respectively.

The lag effect of daily mean temperature on the incidence of BD in Beijing, Tianjin and Hebei indicated that the effect of high temperature was much more significant on lag 5d in Beijing and Hebei, while Tianjin was on lag 7d (Fig. 7). Results of DLNM showed that high temperature increased BD cases by 19% (RR = 1.19, 95% CI: 1.13–1.25) and 15% (RR = 1.15, 95% CI: 1.06–1.24) on lag 5d in Beijing and Hebei, while by 30% (RR = 1.30, 95% CI: 1.18–1.44) on lag 7d in Tianjin, respectively (see Table S2–S3). Each 5 °C rise in temperature caused a 4% (RR = 1.04, 95% CI: 1.02–1.06), 10% (RR = 1.10, 95% CI: 1.06–1.15) and 3% (RR = 1.03, 95% CI: 1.01–1.07) increase after temperature exceeds the threshold in Beijing, Tianjin and Hebei, respectively.

### 3.6 Sensitivity analysis

The sensitivity analysis of this study is to observe and evaluate whether the effect value estimated by DLNM is robust, through changing the parameter settings of the natural cubic spline function and polynomial function in the cross basis of DLNM, including the temperature node selection in the exposure dimension, and the degree of freedom in the lag dimension (*df* = 2∼6). The results (see Fig. S7) showed that the effect value did not change significantly when the relevant parameters were changed, indicating the well selected DLNM model parameters established in this study and the robust model fitting results.

## 4 Discussion

In general, this study is the first time to investigate the influence of daily mean temperature and other meteorological variables on BD in Beijing-Tianjin-Hebei region by using a generalized additive model and a distributed lag non-linear model. The Beijing-Tianjin-Hebei region is located in a warm temperate continental monsoon climate zone, with cold winters, hot summers, and short spring and autumn periods. The four distinct seasons and relatively regular temperature changes explain the obvious seasonality and periodicity in both the daily incidence of BD and the meteorological variables. With the development of social economy in China, sanitation conditions such as water supply facilities and toilets in developed parts have been greatly improved in many areas. As such, BD cases in Beijing and Hebei have decreased in the past two years, although incidence in Tianjin has remained the same (see Fig. S8–S10), probably attributable to person-to-person contact and food contamination [26, 27]. Moreover, the substantial rural-urban migration unique to China may play a role in maintaining BD incidence, as migrants are known to be more vulnerable to infectious diseases [28].

Our study found that high temperature increases the risk of BD, and there is a threshold effect, which means that daily mean temperature has an almost linear relationship with the daily incidence of BD above the threshold of 0 °C. The effect of total temperature on BD also had a turning point of 10 °C: below 10 °C the increase in BD risk was not as pronounced as the increase seen above 10 °C. These are consistent with the results of previous studies with different threshold estimates in different research regions. For example, the study in the southern city of Shenzhen did not detect a temperature threshold [29], whereas another in Beijing reported a temperature threshold of 12.5 °C [30]. The magnitude of temperature influence in this study is also in agreement with previous reports. A study conducted in Jinan found that for every 5 °C increase in the mean temperature, the number of BD cases would increase by 19% [31]. The biological mechanism indicates that the dysentery bacillus is best suited for ambient temperatures of 20–40 °C, with an optimal growth temperature of 37 °C [32]. Therefore, warmer weather is conducive to the reproduction and growth of BD pathogens in the human food chain and water supply, promotes the growth of the bacteria and prolongs the survival time of the bacteria in the environment, increasing the likelihood of human contact with the pathogen. Environmental temperature can also affect specific behavior patterns of the population [33]. For example, warmer weather encourages people to engage in more outdoor activities and increase the contact with others, thereby increasing the transmission channels and facilitating the spread of disease. During warmer weather, people drink more water, which dilutes the gastric juice and weakens its bactericidal function, enhancing the chance of infection from the susceptibility of the population [34].

Analysis of the lag effect manifested that the impact of temperature on BD was highest on lag 5d and lag 6d in the Beijing-Tianjin-Hebei urban agglomeration, and on lag 5d in Beijing and Hebei, while on lag 7d in Tianjin. However, a previous study found the greatest lag effect in the Beijing-Tianjin-Hebei region on lag 4d [20]. This difference in lag day may have been because the previous study was mainly focused on specific spatial cluster areas, and the research period was earlier (2010–2012) and the study subjects were children under 5 years old. The Beijing-Tianjin-Hebei region has a mainly continental climate, with four distinct seasons, and hot, humid summers. However, Tianjin borders the Bohai Sea and has a semi-humid monsoon climate zone in the warm temperate zone [35]. Due to the influence of the Bohai Sea, coastal areas sometimes exhibit oceanic climate characteristics, that is, Tianjin has strong adaptability to temperature adjustment and is more humid, which causes a longer lag effect. In addition, the incidence of BD is closely related to socio-economic factors, such as overcrowded environment and poor health conditions [19, 21]. Differences in climate and socio-economic statuses across the Beijing-Tianjin-Hebei region may explain why the incidence of BD exhibits non-homogeneous spatial characteristics and temporal heterogeneity.

The smooth surface graph suggests that the interaction between temperature and relative humidity has a significant impact on the incidence of BD. The conclusion that a hot, humid weather increases the risk of BD is consistent with previous findings. For example, in Northeast China, the monthly incidence of BD was positively associated with relative humidity and precipitation [8], and other studies also reported similar results in the North China Plain (Beijing, Tianjin, Hebei) [15, 30]. The underlying mechanism may be as follows: high temperature and humidity could provide a suitable environment for promoting bacterial growth and increasing the risk of infection [36]. Etiology studies have also shown that hot and humid conditions increase sensitivity to *Shigella* endotoxin by reducing the function of the reticuloendothelial system [31]. In a high-humidity environment, the temperature felt by the human body is higher than the actual ambient temperature monitored. High relative humidity enhances the effect of temperature, because human reduce their body temperature through sweating and the evaporation of sweat; the latter is inhibited by high relative humidity [37].

Our study also found an inverse relationship between the daily mean wind speed and BD in the Beijing-Tianjin-Hebei region, which is consistent with Liu’s research [31]. Another study in Hunan Province showed that the incidence of BD decreased by 18.54% when the wind speed increased by 1 m/s [14]. This may be because high wind speeds will increase evaporation, reducing the reproduction and survival of pathogens [38]. Moreover, high wind speeds will also decrease contact among people and is thus not conducive to the spread of pathogens. However, our study found that the effect of daily mean wind speed was not significant in Beijing (see Fig. S11–S13). At present, there are no etiological studies on the relationship between wind speed and infectious diseases, therefore, the mechanism through which wind speed affects BD is unclear, and further research is needed. In our study, sunshine duration had no significant effect on BD, nor was there any obvious modification on the effect of temperature. However, a Danish study found that the increase in sunshine duration had a weak positive association with BD cases [38], possibly because sunlight leads to a long-term rise in temperature. But another study declared that sunlight led to greater inhibition of the growth and survival of pathogens [30]. Thus, more research is needed to further study the link between sunlight and the infection.

This study examined not only studies the Beijing-Tianjin-Hebei region, but also Beijing, Tianjin and Hebei separately (see Table S1). We used daily-scale time series data from the past six years (2014 to 2019), which made the analysis more accurate. In addition, this study applied a generalized additive model and stratification to comprehensively and intuitively analyze the effect of meteorological variables and their interaction on the incidence of BD. Based on the analysis of non-linear effects, the application of advanced statistical methods, the distributed lag non-linear model, quantitatively and simultaneously assessed the hysteresis and cumulative effects of temperature. The model was stable and possessed excellent applicability, which could provide the foundation for the establishment of a predictive and early-warning system for the effect of temperature on BD. Meanwhile, some limitations must be acknowledged. The reported data on BD cases were from a passive disease surveillance system, which may be under-reported. Additionally, the occurrence and prevalence of BD are affected by many factors, such as socio-economic conditions, population income, cultural level, residents’ physical fitness and pathogen mutation. Our study was an ecological study that only analyzed the effects of meteorological variables, and the absence of association analysis at individual-level limited the strength of causal inference.

## 5 Conclusions

In conclusion, our research shows that temperature is an important meteorological factor affecting the spread of BD. When the temperature exceeds 10 °C, its increase has a great impact on the incidence of BD. And the effect of temperature can be enhanced by relative humidity and precipitation, which means that a hot and humid environment positively increases the incidence of BD. Moreover, the increase in the incidence of BD caused by high temperature is greatest on lag 5d and lag 6d. This knowledge is beneficial for the prevention and control of BD under different weather conditions, especially hot and humid climates, and the findings are of great significance to the formulation of local strategies for the prevention and reduction of BD in Beijing-Tianjin-Hebei region.

## Abbreviations

BD: Bacillary dysentery
GAM: Generalized additive model
DLNM: Distributed lag non-linear model
RR: Relative risk
CI: Confidence interval
SD: Standard deviation

## Supplementary information

**Additional file 1:** **Table S1** Summary statistic for daily meteorological variables and daily number of BD cases from 2014 to 2019 in Beijing, Tianjin and Hebei. **Figure S1** Relationship curves between meteorological factors and BD in Beijing. **Figure S2** Relationship curves between meteorological factors and BD in Tianjin. **Figure S3** Relationship curves between meteorological factors and BD in Hebei. **Figure S4** The total effect of daily mean temperature in Beijing. **Figure S5** The total effect of daily mean temperature in Tianjin. **Figure S6** The total effect of daily mean temperature in Hebei. **Table S2** The risk of lag effect of different temperatures and lag days on the onset of BD in Beijing, Tianjin and Hebei. **Table S3** The risk of cumulative effect of different temperatures on the onset of BD in Beijing, Tianjin and Hebei. **Figure S7** The sensitivity analysis of effect of daily mean temperature on BD with different related parameters in Beijing-Tianjin-Hebei region. **Figure S8** The time-series distributions of BD and mean temperature in Beijing. **Figure S9** The time-series distributions of BD and mean temperature in Tianjin. **Figure S10** The time-series distributions of BD and mean temperature in Hebei. **Figure S11** The modification and the stratified analysis by other meteorological factors on the effect of mean temperature in Beijing. **Figure S12** The modification and the stratified analysis by other meteorological factors on the effect of mean temperature in Tianjin. **Figure S13** The modification and the stratified analysis by other meteorological factors on the effect of mean temperature in Hebei.

## Declarations

### Availability of data and material

The data that support the findings of this study are available from the data-center of China public health science but restrictions apply to the availability of these data, which were used under license for the current study, and so are not publicly available. Patient data are protected by the China CDC and are unsuitable for public sharing. Interested parties can apply for the data by contacting the data-center of China public health science (https://www.phsciencedata.cn/Share) or email data@chinacdc.cn.
