# Supplementary material for: Effects of daily mean temperature and other meteorological variables on bacillary dysentery in Beijing-Tianjin-Hebei region, China
Source: Environ Health Prev Med. 2022 Mar 19;27:13. doi: 10.1265/ehpm.21-00005 (PMC9251629; doi:10.1265/ehpm.21-00005)
Supplement: Supplementary file 1 — Additional file 1: Table S1 Summary statistic for daily meteorological variables and daily number of BD cases from 2014 to 2019 in Beijing, Tianjin and Hebei. Figure S1 Relationship curves between meteorological factors and BD in Beijing. Figure S2 Relationship curves between meteorological factors and BD in Tianjin. Figure S3 Relationship curves between meteorological factors and BD in Hebei. Figure S4 The total effect of daily mean temperature in Beijing. Figure S5 The total effect of daily mean temperature in Tianjin. Figure S6 The total effect of daily mean temperature in Hebei. Table S2 The risk of lag effect of different temperatures and lag days on the onset of BD in Beijing, Tianjin and Hebei. Table S3 The risk of cumulative effect of different temperatures on the onset of BD in Beijing, Tianjin and Hebei. Figure S7 The sensitivity analysis of effect of daily mean temperature on BD with different related parameters in Beijing-Tianjin-Hebei region. Figure S8 The time-series distributions of BD and mean temperature in Beijing. Figure S9 The time-series distributions of BD and mean temperature in Tianjin. Figure S10 The time-series distributions of BD and mean temperature in Hebei. Figure S11 The modification and the stratified analysis by other meteorological factors on the effect of mean temperature in Beijing. Figure S12 The modification and the stratified analysis by other meteorological factors on the effect of mean temperature in Tianjin. Figure S13 The modification and the stratified analysis by other meteorological factors on the effect of mean temperature in Hebei. [file ehpm-27-013-s001.docx]

**Effects of daily mean temperature and other meteorological variables on bacillary dysentery in Beijing-Tianjin-Hebei region, China**

Qinxue Chang^a*^, Keyun Wang^a*^, Honglu Zhang^a^, Changping Li^a^, Yong Wang^b^, Huaiqi Jing^c^, Shanshan Li^d^, Yuming Guo^d^, Zhuang Cui^a^, Wenyi Zhang^b^

Affiliations

^a^ Department of Epidemiology and Biostatistics, School of Public Health, Tianjin Medical University, Heping District, Tianjin, 300070, P.R. China

^b^ Chinese PLA Center for Disease Control and Prevention, Beijing, 100071, P.R. China

^c^ State Key Laboratory of Infectious Disease Prevention and Control, National Institute for Communicable Disease Control and Prevention, Chinese Center for Disease Control and Prevention, Beijing, 102206, P.R. China

^d^ Department of Epidemiology and Preventive Medicine, School of Public Health and Preventive Medicine, Monash University, Melbourne, Australia

* Qinxue Chang and Keyun Wang contributed equally to this manuscript.

Corresponding author:

Zhuang Cui,

Department of Epidemiology and Biostatistics, School of Public Health, Tianjin Medical University, Heping District, Tianjin, 300070, P.R. China.

E-mail: [cuizhuang@tmu.edu.cn](mailto:cuizhuang@tmu.edu.cn).

Wenyi Zhang,

Chinese PLA Center for Disease Control and Prevention, 20 Dong-Da Street, Fengtai District, Beijing, 100071, P.R. China.

E-mail: zwy0419@126.com.

**Supplemental Materials**

| **Table of Contents** | **Page** |
| --- | --- |
| **Table S1** Summary statistic for daily meteorological variables and daily number of BD cases from 2014 to 2019 in Beijing, Tianjin and Hebei. | 3 |
| **Figure S1-S3** Relationship curves between meteorological variables and BD in Beijing, Tianjin and Hebei. | 4 |
| **Figure S4-S6** The total effect of daily mean temperature in Beijing, Tianjin and Hebei. | 5 |
| **Table S2** The risk of lag effect of different temperatures and lag days on the onset of BD in Beijing, Tianjin and Hebei. | 6 |
| **Table S3** The risk of cumulative effect of different temperatures on the onset of BD in Beijing, Tianjin and Hebei. | 8 |
| **Figure S7** The sensitivity analysis of effect of daily mean temperature on BD with different related parameters in Beijing-Tianjin-Hebei region. | 9 |
| **Figure S8-S10** The time-series distributions of BD and mean temperature in Beijing, Tianjin and Hebei. | 10 |
| **Figure S11-S13** The modification and the stratified analysis by other meteorological factors on the effect of mean temperature in Beijing, Tianjin and Hebei. | 11 |

**Table S1** Summary statistic for daily meteorological variables and daily number of BD cases from 2014 to 2019 in Beijing, Tianjin and Hebei.

|  | Mean ± SD | Minimum | *P*_25_ | *P*_50_ | *P*_75_ | Maximum |
| --- | --- | --- | --- | --- | --- | --- |
| **Beijing** |  |  |  |  |  |  |
| Cases of BD | 22.50$\pm$14.06 | 3 | 12 | 18 | 30 | 77 |
| Mean temperature(°C) | 12.10$\pm$11.45 | -16.6 | 0.8 | 13.7 | 22.7 | 31.0 |
| Relative humidity(%) | 53.37$\pm$18.93 | 9.0 | 38.0 | 53.0 | 69.0 | 96.0 |
| Precipitation(mm) | 1.50$\pm$6.26 | 0 | 0 | 0 | 0 | 166.8 |
| Wind speed(m/s) | 1.71$\pm$0.63 | 0.5 | 1.3 | 1.6 | 2.0 | 5.0 |
| Sunshine duration(h) | 6.75$\pm$3.68 | 0 | 3.9 | 7.7 | 9.5 | 13.2 |
| **Tianjin** |  |  |  |  |  |  |
| Cases of BD | 22.42$\pm$13.80 | 0 | 11 | 18 | 34 | 63 |
| Mean temperature(°C) | 13.66$\pm$11.19 | -14.8 | 2.4 | 15.0 | 24.1 | 32.9 |
| Relative humidity(%) | 57.51$\pm$18.30 | 13.0 | 44.0 | 58.0 | 72.0 | 98.0 |
| Precipitation(mm) | 1.47$\pm$6.82 | 0 | 0 | 0 | 0 | 175.5 |
| Wind speed(m/s) | 2.41$\pm$0.92 | 0.7 | 1.7 | 2.2 | 2.9 | 6.7 |
| Sunshine duration(h) | 6.85$\pm$3.83 | 0 | 4.0 | 7.8 | 9.9 | 13.6 |
| **Hebei** |  |  |  |  |  |  |
| Cases of BD | 22.18$\pm$14.84 | 0 | 11 | 18 | 30 | 95 |
| Mean temperature(°C) | 11.22$\pm$11.45 | -18.0 | -0.1 | 12.8 | 21.8 | 30.3 |
| Relative humidity(%) | 57.53$\pm$15.85 | 17 | 45 | 58 | 70.3 | 93 |
| Precipitation(mm) | 1.39$\pm$4.19 | 0 | 0 | 0 | 0.8 | 97.6 |
| Wind speed(m/s) | 2.21$\pm$0.69 | 0.9 | 1.7 | 2.1 | 2.6 | 5.8 |
| Sunshine duration(h) | 6.86$\pm$3.20 | 0 | 4.6 | 7.5 | 9.3 | 13.3 |
| Abbreviations: SD, standard deviation; *P_x_*, xth percentile. | | | | | | |


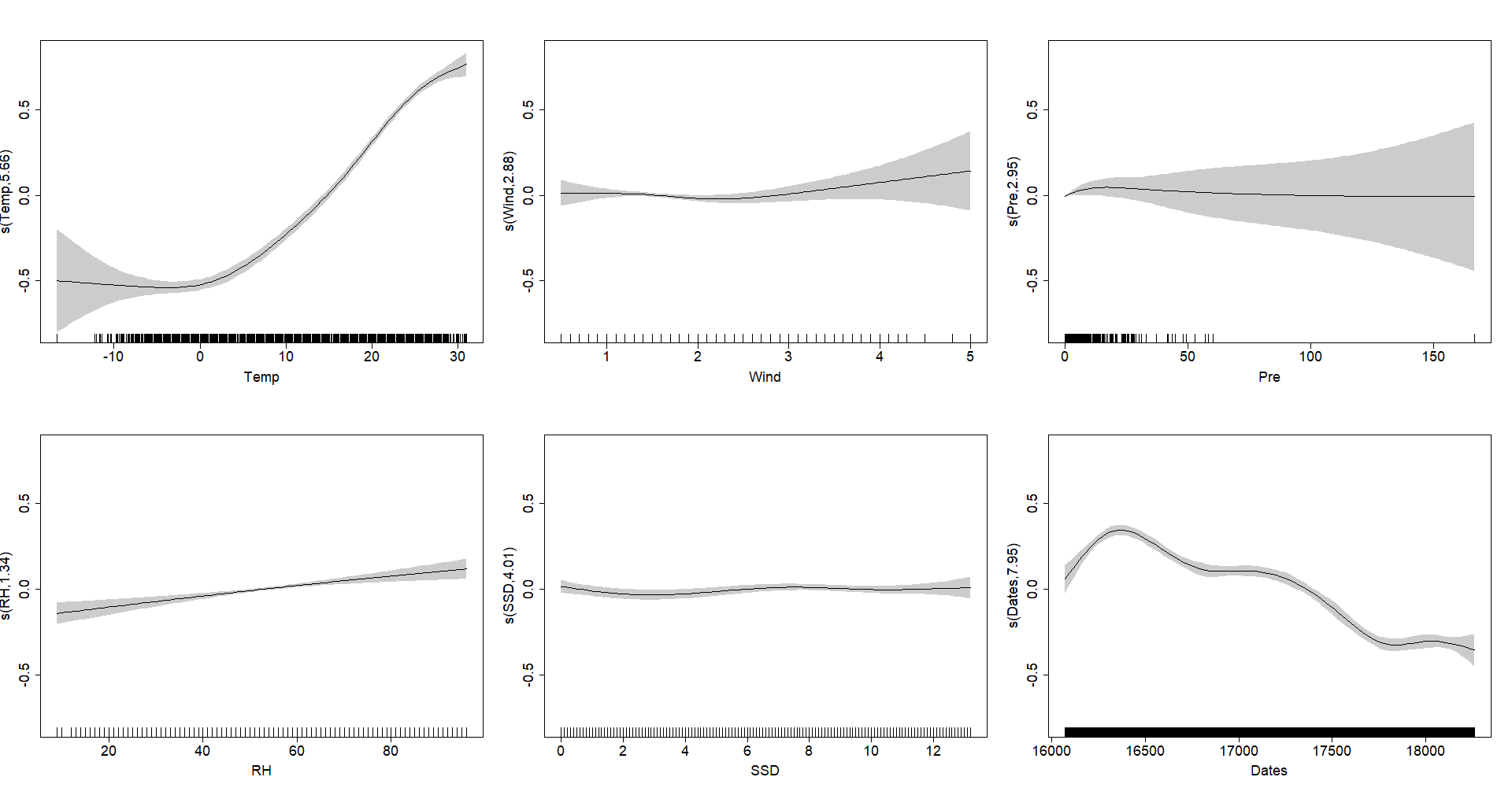


**Figure S1** Relationship curves between meteorological factors and BD in Beijing.


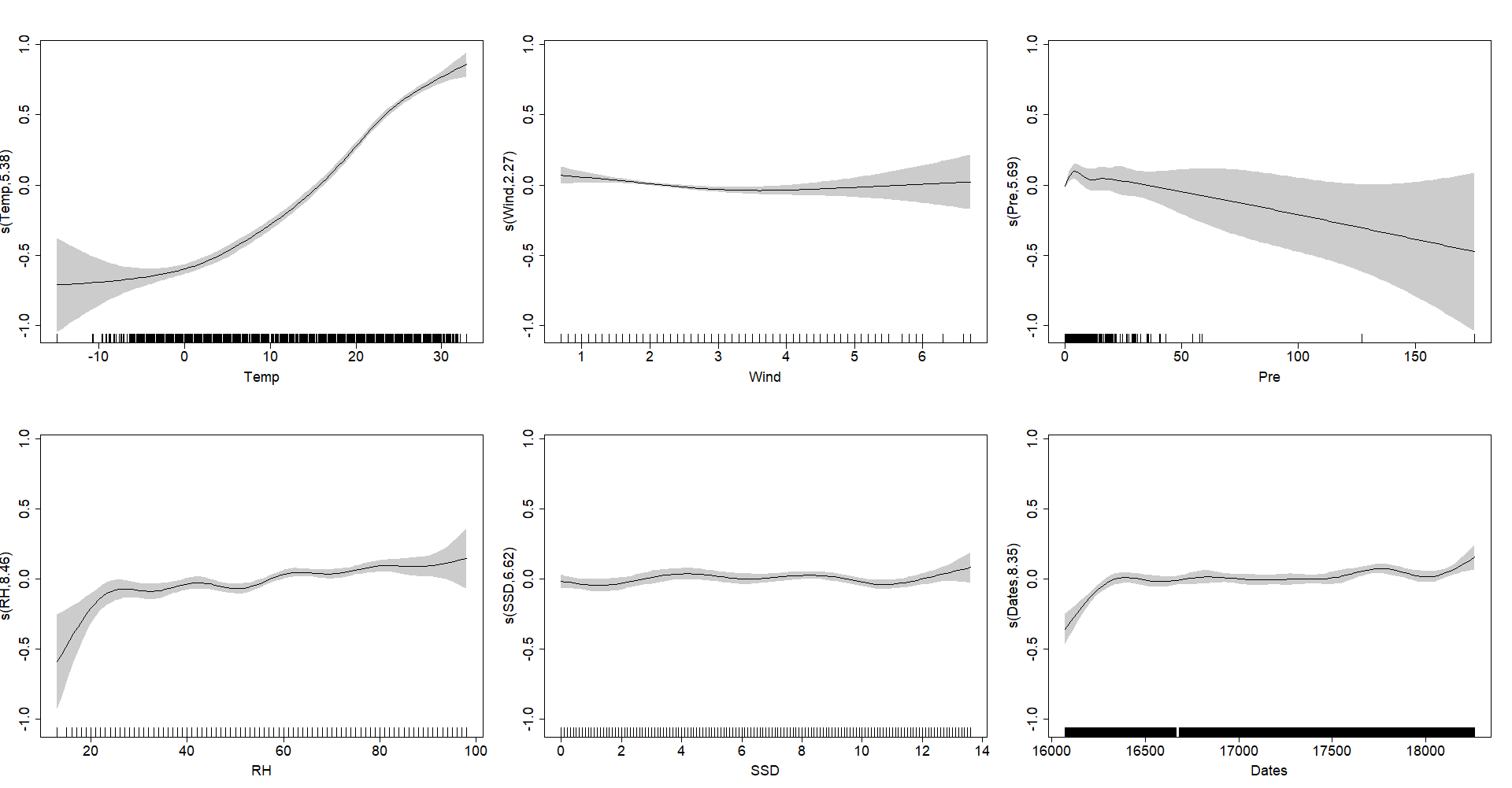


**Figure S2** Relationship curves between meteorological factors and BD in Tianjin.


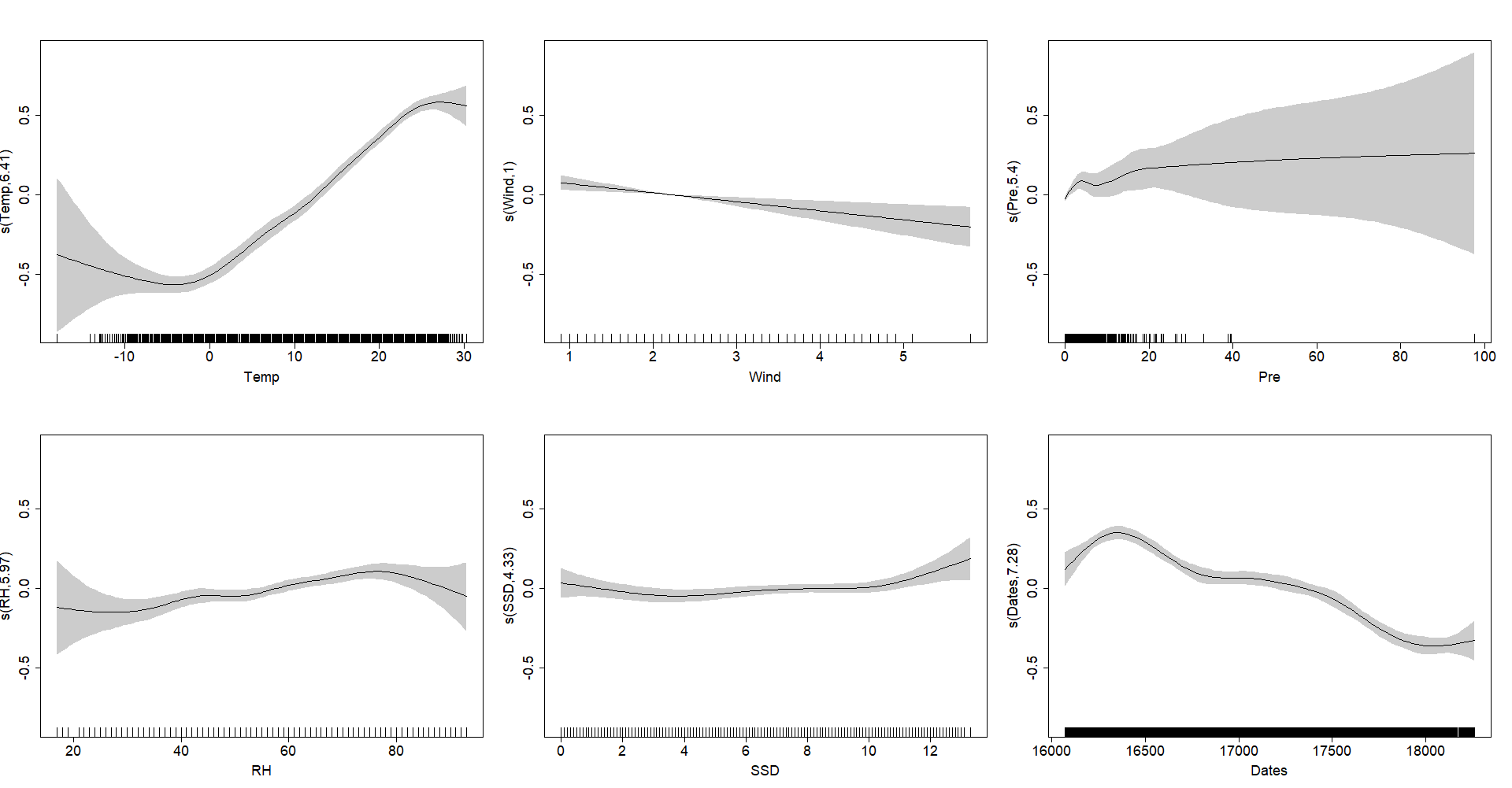


**Figure S3** Relationship curves between meteorological factors and BD in Hebei.


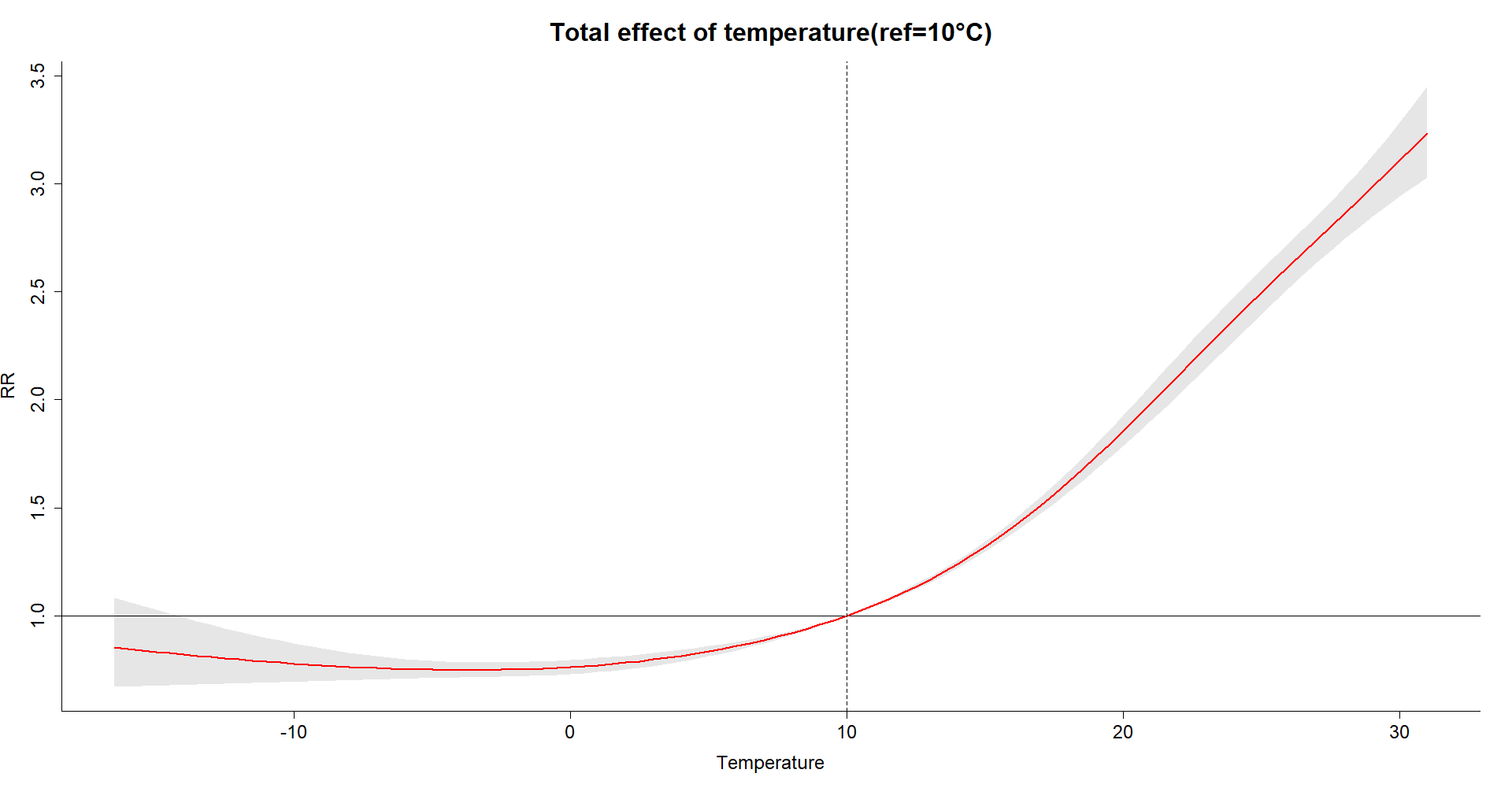


**Figure S4** The total effect of daily mean temperature in Beijing.


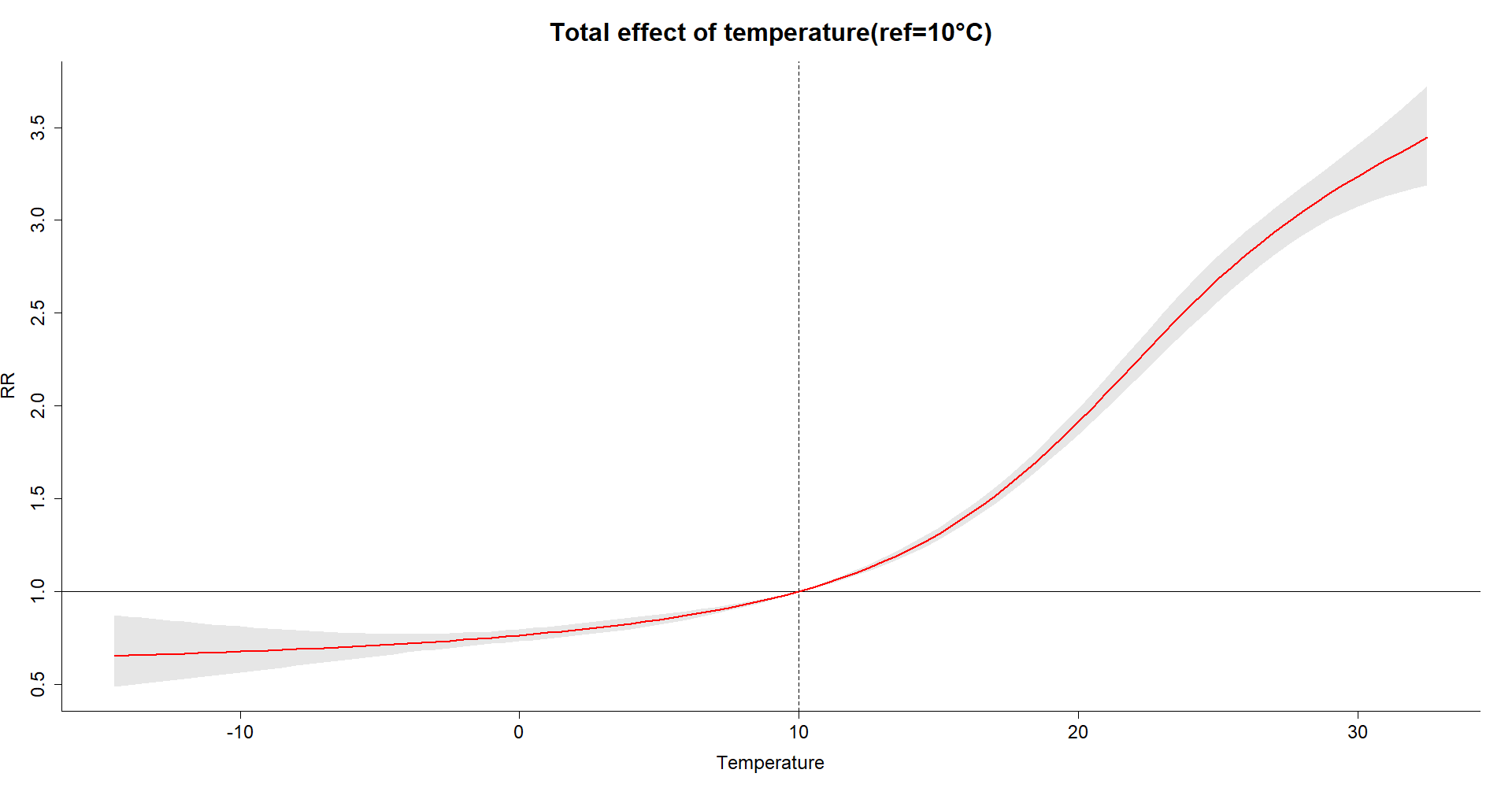


**Figure S5** The total effect of daily mean temperature in Tianjin.


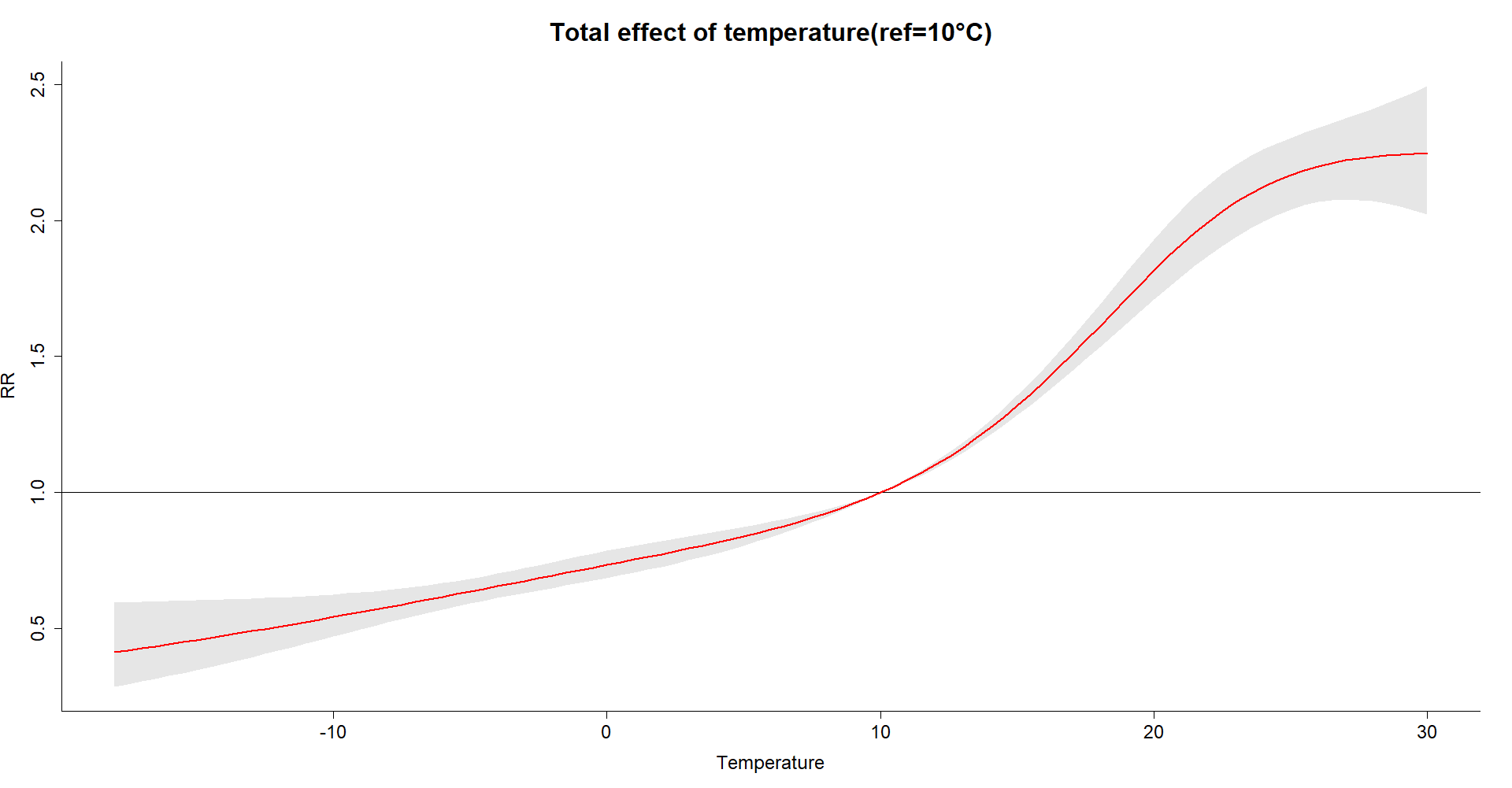


**Figure S6** The total effect of daily mean temperature in Hebei.

**Table S2** The risk of lag effect of different temperatures and lag days on the onset of BD in Beijing, Tianjin and Hebei.

|  | **Lag0** | **Lag1** | **Lag2** | **Lag3** | **Lag4** | **Lag5** | **Lag6** | **Lag7** |
| --- | --- | --- | --- | --- | --- | --- | --- | --- |
| **Beijing** |  |  |  |  |  |  |  |  |
| *P_2.5_*: -7℃ | 1.020  (0.896-1.161) | 1.001  (0.943-1.063) | 0.974  (0.899-1.056) | 0.948  (0.891-1.009) | 0.929  (0.873-0.989) | 0.925  (0.853-1.003) | 0.942  (0.887-1.001) | 0.991  (0.870-1.128) |
| *P_25_*: 1℃ | 1.044  (0.958-1.138) | 0.978  (0.940-1.018) | 0.954  (0.905-1.006) | 0.954  (0.916-0.994) | 0.965  (0.926-1.006) | 0.972  (0.921-1.025) | 0.961  (0.924-1) | 0.920  (0.844-1.003) |
| *P_50_*: 13.5℃ | 1.000  (0.972-1.028) | 1.018  (1.005-1.031) | 1.026  (1.008-1.043) | 1.026  (1.013-1.039) | 1.024  (1.010-1.037) | 1.023  (1.006-1.04) | 1.028  (1.015-1.041) | 1.043  (1.015-1.072) |
| *P_75_*: 22.5℃ | 1.099  (1.011-1.194) | 1.074  (1.037-1.114) | 1.077  (1.026-1.131) | 1.095  (1.055-1.136) | 1.118  (1.078-1.159) | 1.133  (1.080-1.189) | 1.129  (1.089-1.171) | 1.094  (1.009-1.186) |
| *P_97.5_*: 28.5℃ | 1.185  (1.076-1.305) | 1.113  (1.069-1.158) | 1.102  (1.042-1.165) | 1.126  (1.079-1.175) | 1.162  (1.116-1.211) | **1.187**  **(1.125-1.253)** | 1.175  (1.127-1.224) | 1.103  (1.007-1.208) |
| **Tianjin** |  |  |  |  |  |  |  |  |
| *P_2.5_*: -5℃ | 1.056  (0.923-1.209) | 1.007  (0.946-1.071) | 0.984  (0.905-1.070) | 0.974  (0.913-1.038) | 0.965  (0.905-1.029) | 0.946  (0.870-1.03) | 0.907  (0.853-0.965) | 0.841  (0.734-0.962) |
| *P_25_*: 2.5℃ | 1.073  (0.995-1.156) | 1.009  (0.975-1.045) | 0.982  (0.938-1.028) | 0.974  (0.940-1.009) | 0.972  (0.939-1.007) | 0.964  (0.921-1.009) | 0.937  (0.905-0.969) | 0.880  (0.816-0.948) |
| *P_50_*: 15℃ | 0.965  (0.924-1.007) | 1.006  (0.987-1.026) | 1.027  (1.002-1.054) | 1.035  (1.015-1.055) | 1.038  (1.018-1.058) | 1.044  (1.018-1.071) | 1.062  (1.042-1.083) | 1.103  (1.056-1.151) |
| *P_75_*: 24℃ | 1.014  (0.926-1.11) | 1.066  (1.025-1.108) | 1.097  (1.041-1.155) | 1.115  (1.071-1.160) | 1.129  (1.086-1.174) | 1.149  (1.091-1.210) | 1.185  (1.139-1.232) | 1.250  (1.144-1.365) |
| *P_97.5_*: 30℃ | 1.081  (0.975-1.199) | 1.109  (1.063-1.158) | 1.123  (1.059-1.192) | 1.132  (1.082-1.184) | 1.144  (1.095-1.195) | 1.169  (1.103-1.238) | 1.218  (1.165-1.272) | **1.304**  **(1.181-1.439)** |
| **Hebei** |  |  |  |  |  |  |  |  |
| *P_2.5_*: -8.5℃ | 1.112  (0.911-1.356) | 0.927  (0.847-1.014) | 0.894  (0.789-1.012) | 0.935  (0.850-1.028) | 0.996  (0.907-1.095) | 1.014  (0.896-1.147) | 0.925  (0.844-1.013) | 0.709  (0.581-0.865) |
| *P_25_*: 0℃ | 0.996  (0.873-1.136) | 0.93  (0.876-0.986) | 0.928  (0.856-1.005) | 0.961  (0.904-1.022) | 1.003  (0.943-1.066) | 1.022  (0.944-1.107) | 0.988  (0.932-1.048) | 0.878  (0.771-1.000) |
| *P_50_*: 13℃ | 1.019  (0.987-1.052) | 1.024  (1.010-1.038) | 1.024  (1.005-1.044) | 1.021  (1.007-1.036) | 1.017  (1.003-1.031) | 1.014  (0.995-1.033) | 1.014  (1.000-1.028) | 1.019  (0.988-1.051) |
| *P_75_*: 22℃ | 1.097  (0.973-1.235) | 1.075  (1.024-1.129) | 1.081  (1.010-1.157) | 1.101  (1.046-1.158) | 1.120  (1.066-1.176) | 1.123  (1.051-1.201) | 1.098  (1.044-1.154) | 1.031  (0.921-1.154) |
| *P_97.5_*: 27.5℃ | 1.149  (0.997-1.324) | 1.095  (1.035-1.159) | 1.094  (1.008-1.187) | 1.119  (1.053-1.190) | 1.147  (1.084-1.214) | **1.151**  **(1.064-1.244)** | 1.105  (1.042-1.172) | 0.992  (0.870-1.133) |
| Abbreviations: *P_x_*, xth percentile; Lagy, the yth day of lag. | | | | | | | | |

**Table S3** The risk of cumulative effect of different temperatures on the onset of BD in Beijing, Tianjin and Hebei.

|  | *P_2.5_* | *P_25_* | *P_50_* | *P_75_* | *P_97.5_* |
| --- | --- | --- | --- | --- | --- |
| **Beijing** | 0.757  (0.705-0.812) | 0.771  (0.738-0.804) | 1.202  (1.185-1.219) | 2.179  (2.085-2.277) | 2.924  (2.797-3.056) |
| **Tianjin** | 0.71  (0.653-0.772) | 0.8  (0.768-0.832) | 1.31  (1.277-1.344) | 2.537  (2.423-2.657) | 3.237  (3.074-3.408) |
| **Hebei** | 0.57  (0.51-0.638) | 0.735  (0.686-0.786) | 1.164  (1.145-1.183) | 1.997  (1.871-2.132) | 2.228  (2.075-2.391) |


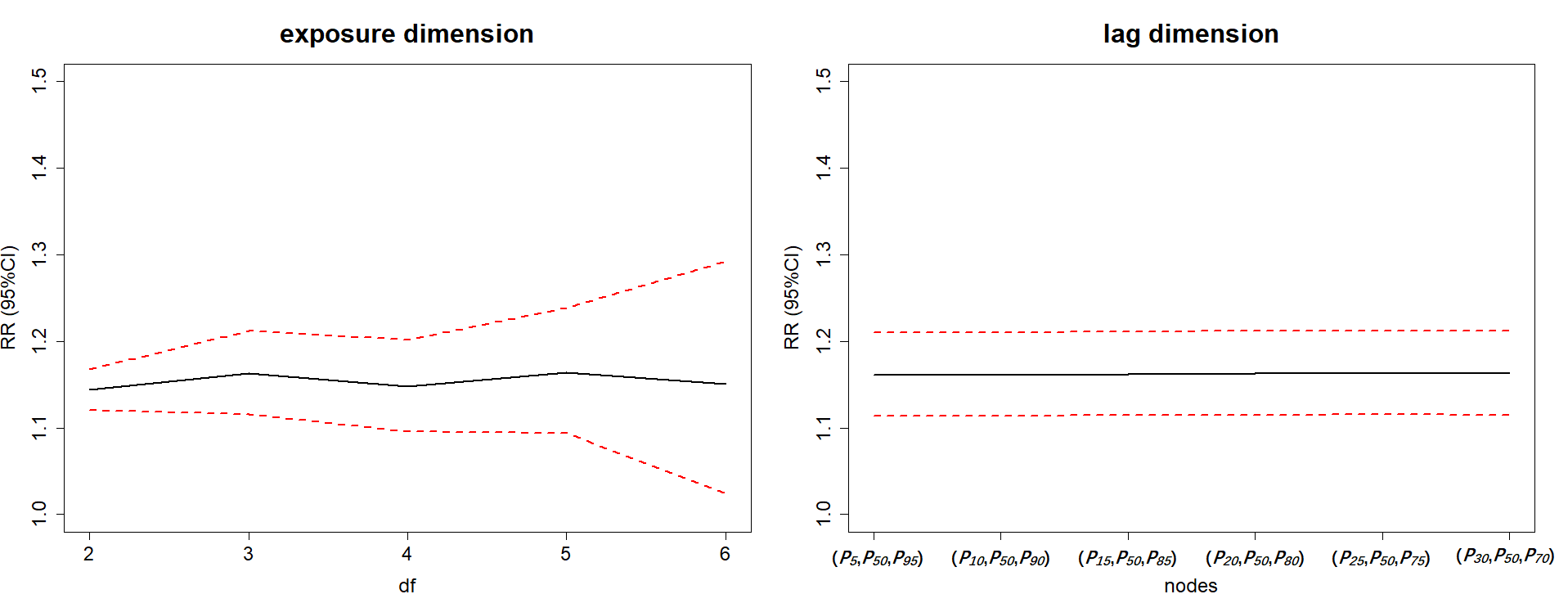
**Figure S7** The sensitivity analysis of effect of daily mean temperature on BD with different related parameters in Beijing-Tianjin-Hebei region. *P_x_*, xth percentile of temperature.


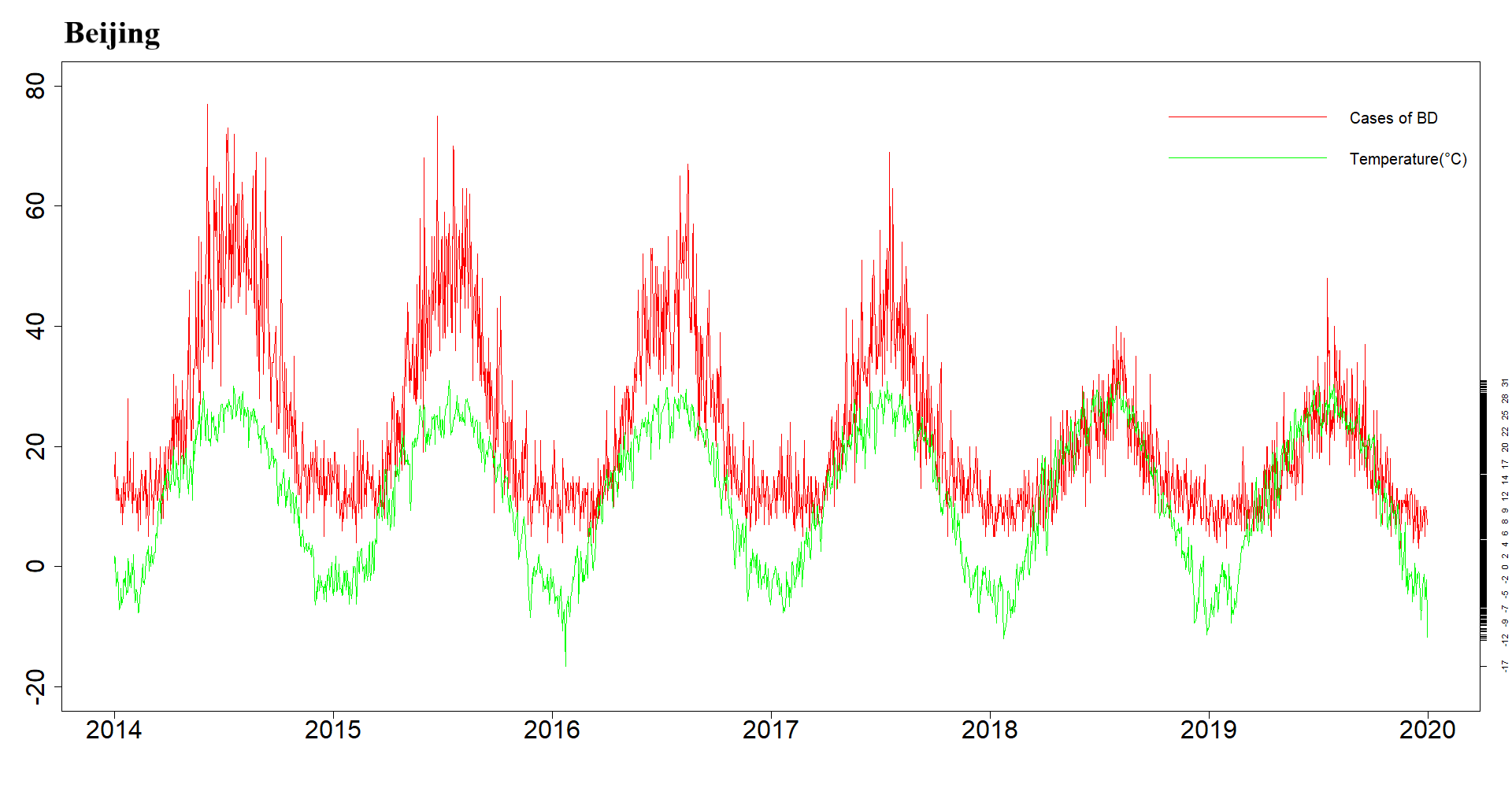


**Figure S8** The time-series distributions of BD and mean temperature in Beijing.


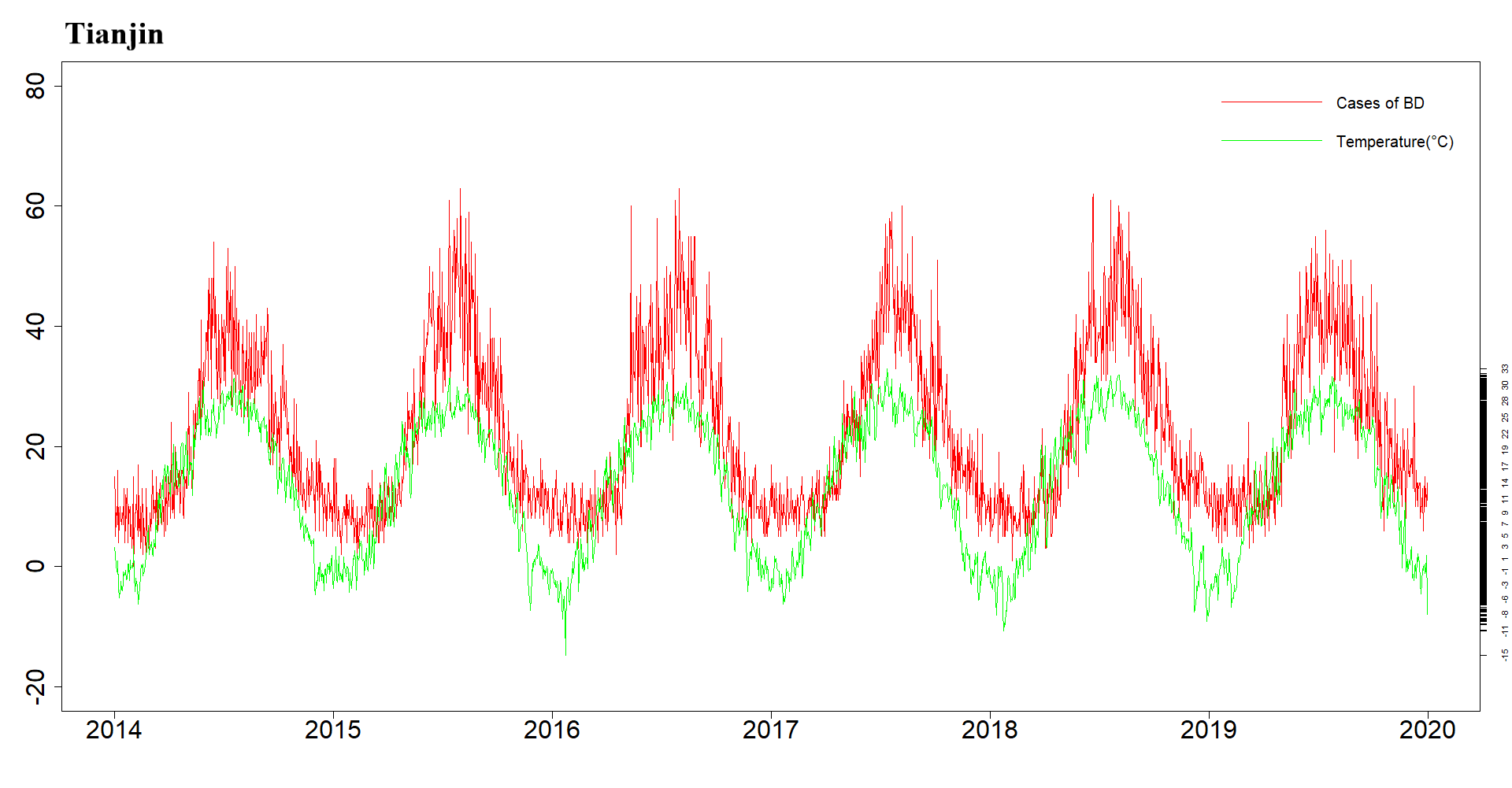


**Figure S9** The time-series distributions of BD and mean temperature in Tianjin.


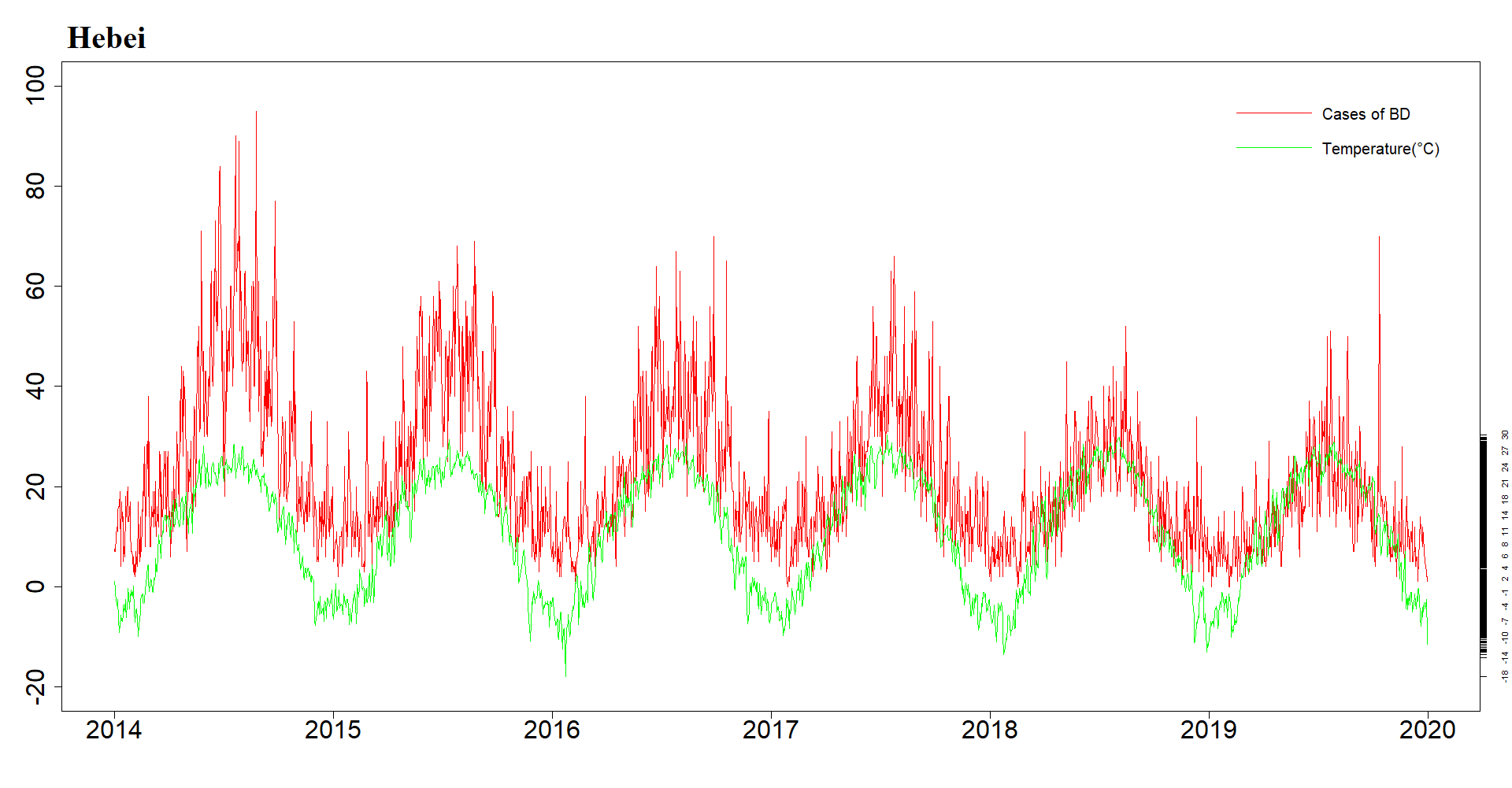


**Figure S10** The time-series distributions of BD and mean temperature in Hebei.


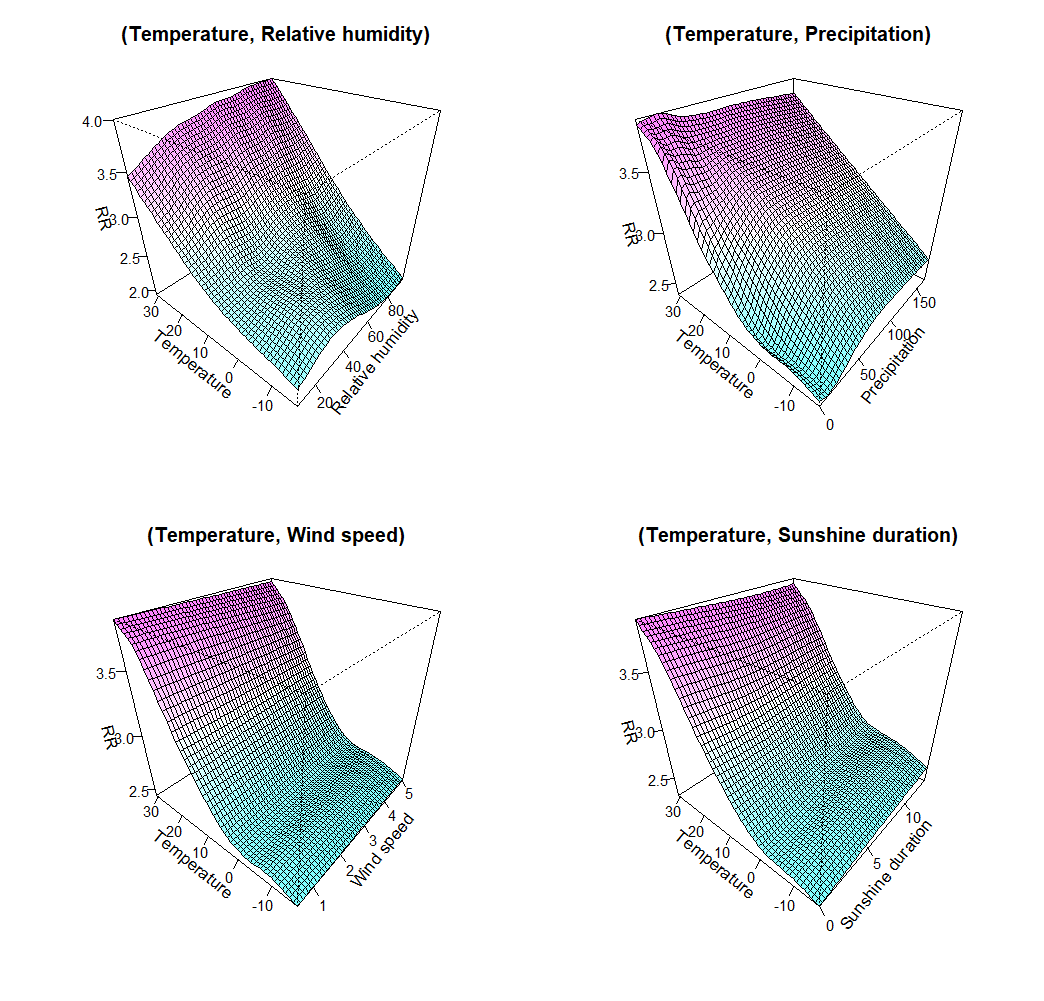

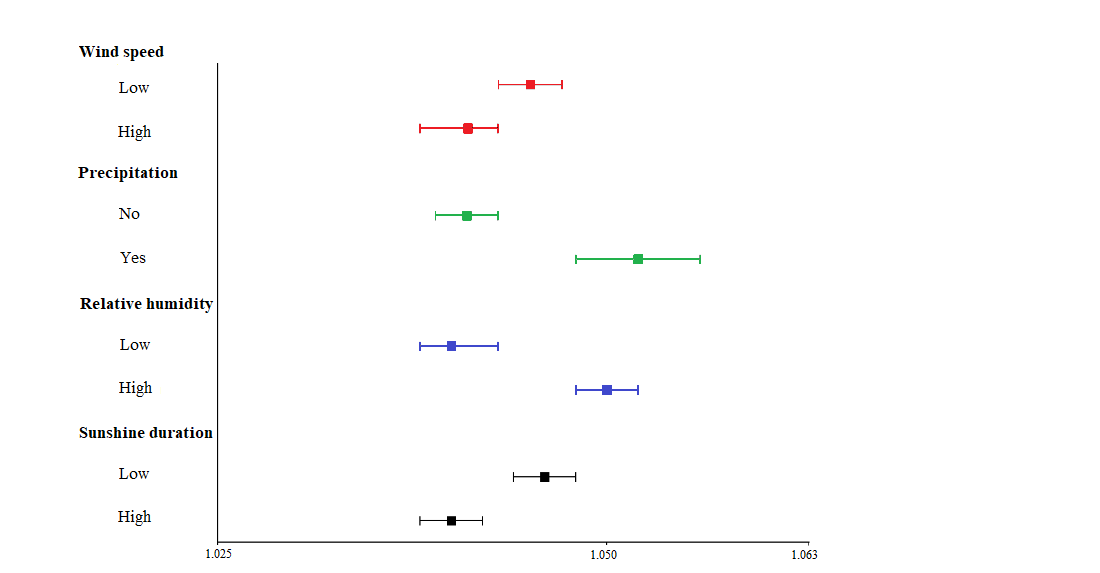


**Figure S11** The modification and the stratified analysis by other meteorological factors on the effect of mean temperature in Beijing.


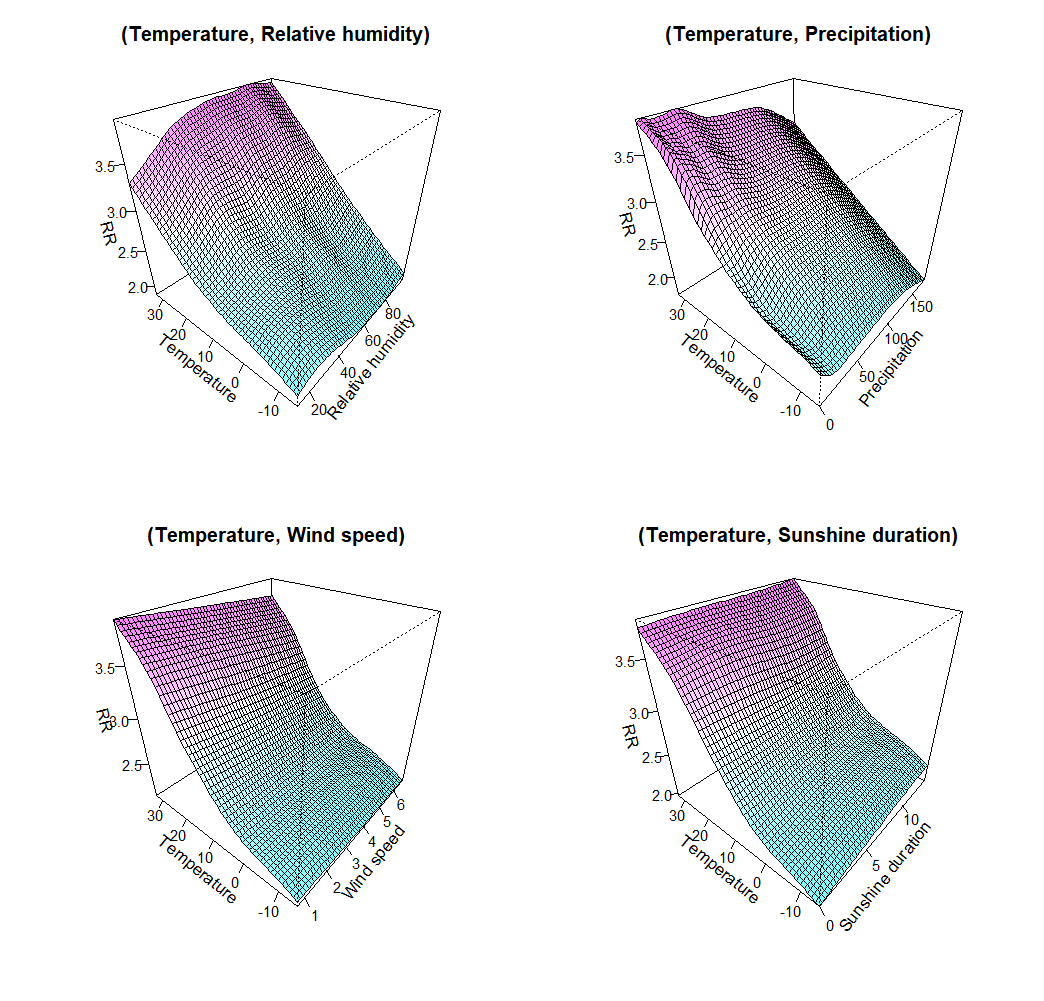

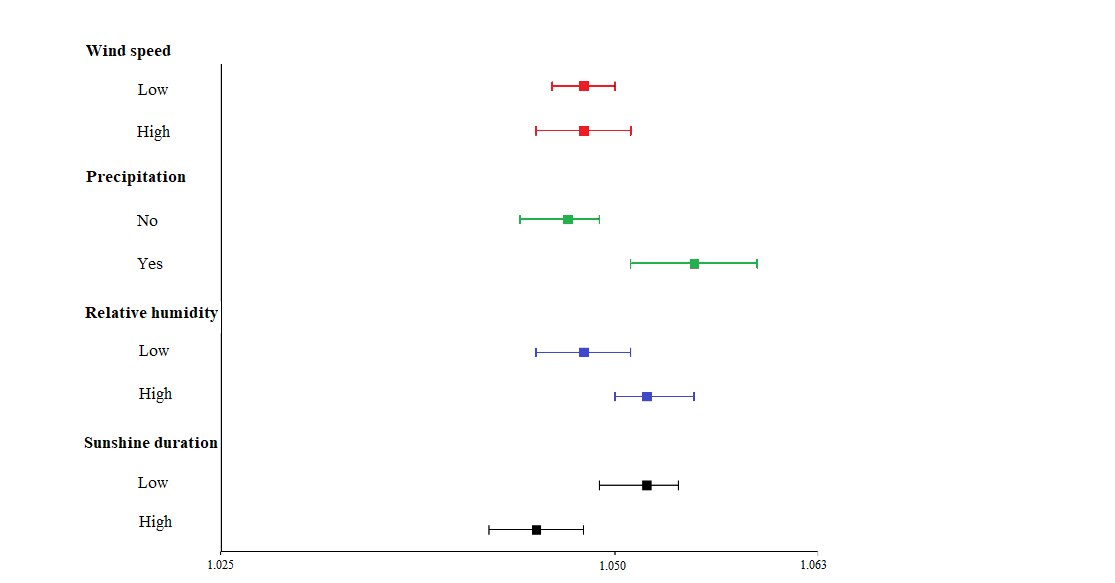


**Figure S12** The modification and the stratified analysis by other meteorological factors on the effect of mean temperature in Tianjin.


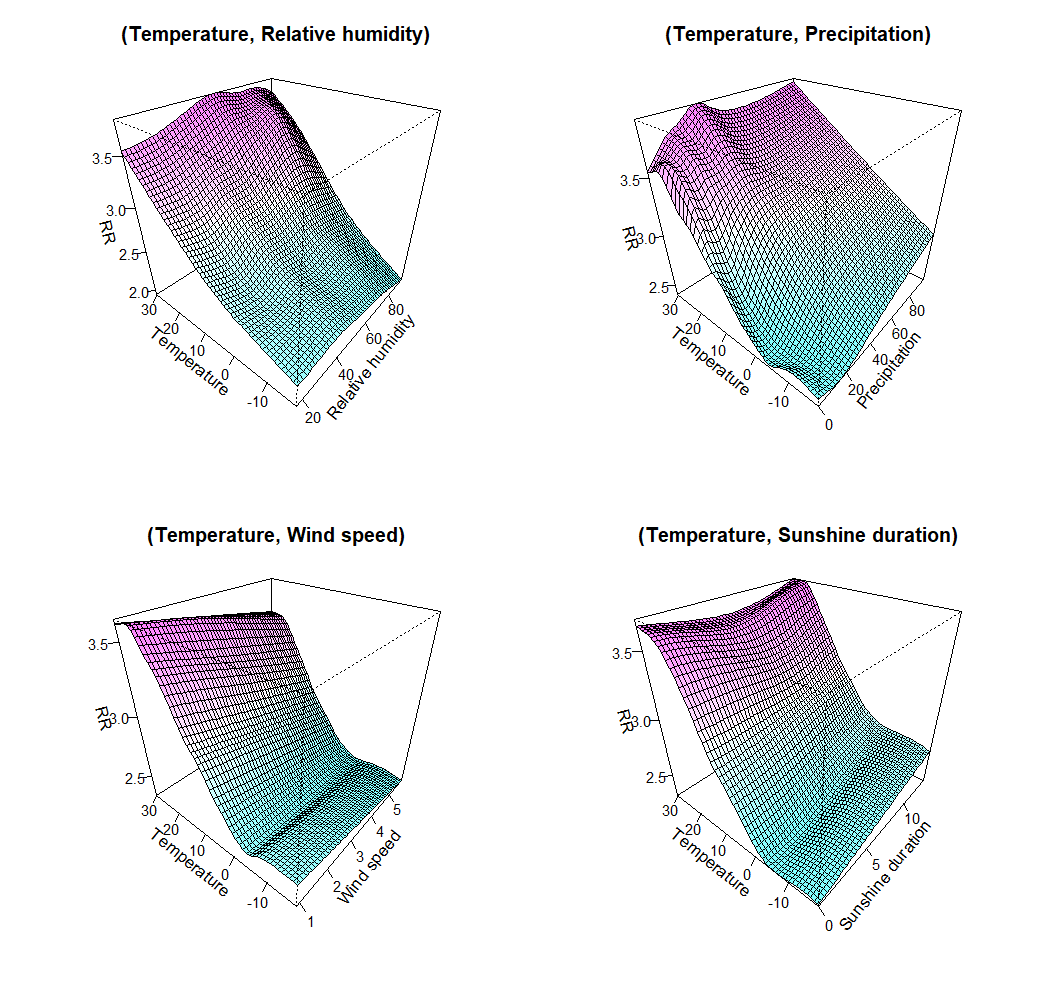

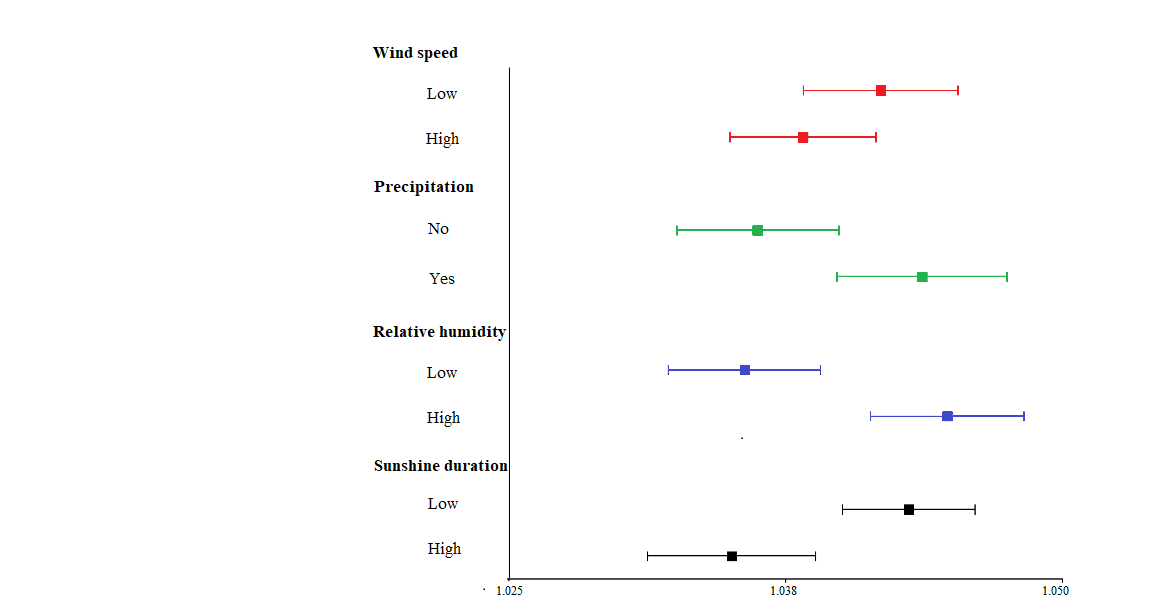


**Figure S13** The modification and the stratified analysis by other meteorological factors on the effect of mean temperature in Hebei.
